# Reprogramming *Mycobacterium tuberculosis* CRISPR System for Gene Editing and Genome-wide RNA Interference Screening

**DOI:** 10.1016/j.gpb.2021.01.008

**Published:** 2021-12-16

**Authors:** Khaista Rahman, Muhammad Jamal, Xi Chen, Wei Zhou, Bin Yang, Yanyan Zou, Weize Xu, Yingying Lei, Chengchao Wu, Xiaojian Cao, Rohit Tyagi, Muhammad Ahsan Naeem, Da Lin, Zeshan Habib, Nan Peng, Zhen F. Fu, Gang Cao

**Affiliations:** 1 State Key Laboratory of Agricultural Microbiology, Huazhong Agricultural University, Wuhan 430070, China; 2 College of Veterinary Medicine, Huazhong Agricultural University, Wuhan 430070, China; 3 Bio-Medical Center, Huazhong Agricultural University, Wuhan 430070, China; 4 Key Laboratory of Preventive Veterinary Medicine in Hubei Province, the Cooperative Innovation Center for Sustainable Pig Production, Wuhan 430070, China

**Keywords:** *Mycobacterium tuberculosis*, Type III-A CRISPR system, Gene editing, Gene interference, Genome-wide RNAi screening

## Abstract

***Mycobacterium tuberculosis*** is the causative agent of tuberculosis (TB), which is still the leading cause of mortality from a single infectious disease worldwide. The development of novel anti-TB drugs and vaccines is severely hampered by the complicated and time-consuming genetic manipulation techniques for *M. tuberculosis.* Here, we harnessed an endogenous type III-A CRISPR/Cas10 system of *M. tuberculosis* for efficient **gene editing** and RNA interference (RNAi). This simple and easy method only needs to transform a single mini-CRISPR array plasmid, thus avoiding the introduction of exogenous protein and minimizing proteotoxicity. We demonstrated that *M. tuberculosis* genes can be efficiently and specifically knocked in/out by this system as confirmed by DNA high-throughput sequencing. This system was further applied to single- and multiple-gene RNAi. Moreover, we successfully performed **genome-wide RNAi screening** to identify *M. tuberculosis* genes regulating *in vitro* and intracellular growth. This system can be extensively used for exploring the functional genomics of *M. tuberculosis* and facilitate the development of novel anti-TB drugs and vaccines.

## Introduction

*Mycobacterium tuberculosis* is the etiological agent of tuberculosis (TB), and is currently the deadliest pathogen which ranks above HIV and causes approximately 1.3 million deaths and 10 million new cases globally [1]. The current treatment for TB comprises the extensive administration of antibiotics, which often leads to the emergence of extensively drug-resistant bacteria [2]. Meanwhile, the intake of rifampicin, isoniazid, or pyrazinamide can adversely affect the composition of the gut microbiota [3], [4]. Functional genomic analysis of *M. tuberculosis* and elucidation of the molecular mechanism underlying TB pathophysiology are crucial for the identification of novel drugs and candidate vaccine genes. However, advances in *M. tuberculosis* functional genomic studies are greatly impeded by inefficient tools for gene editing and silencing. Conventional methods for gene editing are laborious and time-consuming [5]. Recently, oligonucleotide-mediated recombineering followed by Bxb1 integrase targeting (ORBIT) has been developed for gene editing in *M. tuberculosis.* ORBIT is based on the integration of a targeting oligonucleotide into the genome through homologous recombination mediated by the phage Che9c RecT annealase. However, this method needs the transformation of a single-stranded DNA (ssDNA) probe along with a nonreplicating plasmid [6].

The clustered regularly interspaced short palindromic repeats (CRISPR) and associated proteins (Cas) system has been extensively used for genome editing in bacteria [7]. CRISPR/Cas9 systems from *Streptococcus pyogenes* and *S. thermophilus* have been used for sequence-specific transcriptional repression in *M. tuberculosis* [8], [9], [10]. Rock et al. [11] screened a group of Cas9 proteins for gene silencing in *M. tuberculosis* and found that dCas9 from *S. thermophilus* is efficient for gene silencing in *M. tuberculosis* and has low proteotoxicity. However, efforts to use CRISPR/Cas9 systems to disrupt genes in *M. bovis* and *M. smegmatis* have failed [12], [13]. To date, none of these methods have been applied to simultaneously silence multiple genes in *M. tuberculosis*.

The type III CRISPR/Cas system is presented in approximately 75% of archaea and 40% of bacteria, including pathogenic *Mycobacterium* and *Staphylococcus* species [14]. This system is further classified into four subtypes that are characterized by the Csm complex (type III-A and D) or the Cmr complex (type III-B and C) [15]. The initial transcription of a CRISPR array yields an immature long transcript known as precursor CRISPR-RNA (pre-crRNA), which is processed by Cas6 along with other effector proteins to produce mature crRNAs. A mature crRNA harbors 8-nt tag at its 5′-end known as the “crRNA-tag” which is of pivotal importance in autoimmunity scenario [16]. Once a mature crRNA is generated, a Csm/Cmr complex interacts with the crRNA to form a crRNA ribonucleoprotein (crRNP) complex [17].

The type III CRISPR/Cas system possesses robust RNA cleavage and transcription-dependent ssDNA cutting capabilities, thus efficiently providing immunity against invading genomic elements [18]. RNA cleavage is mediated by Csm3/Cmr4 or Csm6/Csx1, and Cas10/Csm1 cleaves ssDNA [17], [19]. Upon activation, Cas10 cleaves ssDNA [20], [21] and generates cyclic oligoadenylate (CoA) signaling molecules from ATP, which further act as an activator of the RNA-targeting proteins (Csm6 family nucleases) [22], [23].

The type III CRISPR/Cas system can be reprogrammed for RNA targeting and genome editing in *S. thermophilus* and *Sulfolobus islandicus* [24], [25], [26] and for chromosomal targeting to achieve large-scale genomic deletion and alteration in *Staphylococcus aureus* [27]. Bari et al. [28] used this system to edit the DNA of virulent *Staphylococcal* phages. Wei et al. [29] reported that *Mycobacterium* species contain a typical feature of type III-A systems and are highly active against the invading DNA. Moreover, *M. tuberculosis* CRISPR systems effectively target RNA and DNA upon expression in *Escherichia coli* [25], [30]. However, these systems have not been used for gene editing, RNA interference (RNAi), and genome-wide RNAi screening for *M. tuberculosis.*

Therefore, the aim of this study is to employ an endogenous type III-A CRISPR system of *M. tuberculosis* and use it to develop a versatile and robust tool for efficient gene editing, RNAi, and genome-wide RNAi screening. We demonstrated that this system can be used for robust and facile gene knock-in/knockout processes. Moreover, this system can be used for single- and multiple-gene RNAi to precisely dissect the functions of specific genes. Furthermore, we applied this system for the genome-scale RNAi screening of growth-regulating genes. This “killing two birds with one stone” tool may facilitate the genetic and functional studies of *M. tuberculosis* and pave the way for the development of anti-TB drugs and potent vaccines.

## Results

### Comparison of the type III-A CRISPR system in mycobacteria

**Figure 1:**
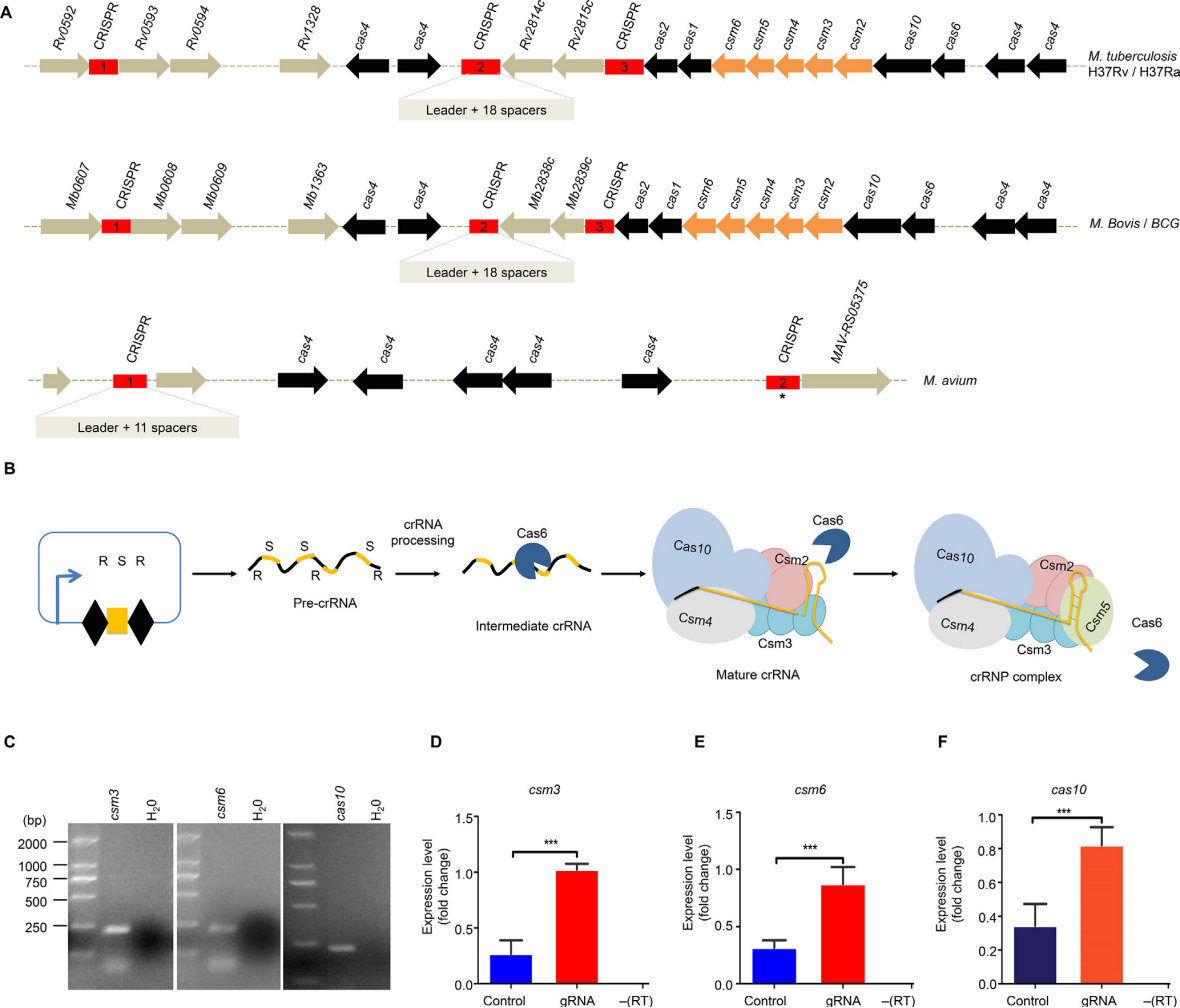
**Type III-A CRISPR/Cas10 system ofmycobacteria** **A.** Type III-A CRISPR/Cas10 system in *M. tuberculosis*, *M. bovis*, and *M. avium*. * indicates that the CRISPR locus 2 of the *M. avium* CRISPR system is characterized. **B.** Schematic shows crRNA biogenesis by the type III-A CRISPR crRNP complex. This crRNP complex then cleaves DNA through a transcription-dependent process and RNA through a transcription-independent process. **C.** Expression of *csm3*, *csm6*, and *cas10* in *M. tuberculosis* detected by qRT-PCR. **D.**–**F.** qRT-PCR identification of the expression of *csm3*, *csm6*, and *cas10* in strains transformed with the mini-CRISPR array (containing gRNA) and empty vector (control), respectively. –(RT) indicates a negative control to ensure that no genomic DNA contamination occurred. Student’s *t*-test (***, *P* < 0.001). R, repeat; S, spacer; crRNP, crRNA ribonucleoprotein.

The CRISPR systems of three different species of *Mycobacterium* were explored and compared using the NCBI BLAST, Mycobrowser (https://mycobrowser.epfl.ch/), and KEGG genome database (http://www.genome.jp/). The CRISPR systems of *M. tuberculosis* H37Rv and H37Ra comprise three CRISPR loci in association with 10 *cas* genes, namely *cas2*, *cas1*, *csm6*, *csm5*, *csm4*, *csm3*, *csm2*, *cas10*, *cas6*, and *cas4* (Figure 1A). The CRISPR locus 1 of *M. tuberculosis* contains only one spacer flanked by two repeats and is distant from the other two CRISPR loci and their associated *cas* genes. Loci 2 and 3 are interspaced by two transposase genes belonging to the insertion sequence (*IS6110*) gene family followed by a cluster of nine consecutive *cas* genes. Loci 2 and 3 contain 18 and 24 spacers, respectively, and have well-characterized leader sequences at the 5ʹ-ends. Similarly, the CRISPR systems of *M. bovis* and *M. bovis* BCG have the same number and length of repeats and spacers as that of *M. tuberculosis* (Figure 1A). However, the CRISPR system of *M. avium* has one uncharacterized and one fully characterized long CRISPR locus associated with five c*as4* genes, with the former containing 11 spacers (Figure 1A).

Like other type III CRISPR systems, *M. tuberculosis* type III-A CRISPR system is transcribed into a polycistronic mRNA which is further processed into many mature crRNAs. The mature crRNAs are of uniform length and will not be subjected to further processing at 3′-end after Cas6 cleaves the repeat region at 3′-end (Figure 1B) [29]. To investigate the expression of type III-A CRISPR system genes in *M. tuberculosis*, we performed qRT-PCR on three functional candidate genes (*csm3*, *csm6*, and *cas10*). We found the constitutive expression of these genes in *M. tuberculosis* (Figure 1C). To determine whether the expression of the CRISPR array affects the expression of these *cas* genes in *M. tuberculosis*, we constructed an expression plasmid named pMV-261-crRNA. This plasmid contains two repeat sequences (36 bp each) from CRISPR locus 3 (Figure S1A). Then, a 40-bp gRNA was cloned into the *Bbs*I site flanked by the repeats in the pMV-261-crRNA plasmid and transformed into the bacilli. Notably, the expression levels of the three genes increased by more than 50% when *M. tuberculosis* was transformed with the plasmid expressing the mini-CRISPR array under the control of *Psmyc* promoter when compared to the control strain (Figure 1D–F). Sanger sequencing was performed on the qRT-PCR amplicons to confirm the sequences of the three genes (Figure S1B). Overall, these results suggest that the expression of *cas* genes was enhanced upon the expression of the CRISPR array.

Type III-A CRISPR systems synthesize CoAs that activate Csm6 to target cellular RNA and thus confers robust host immunity against foreign invading elements [31]. These systems can cut ssDNA [20]. Therefore, we harnessed this system for *M. tuberculosis* self-DNA targeting by designing a 40-bp gRNA complementary to the coding strand of the *gyrase A* (*gyrA*) gene (Figure S1C). A homology-directed repair (HDR) template containing the 400-bp upstream and downstream regions of the *gyrA* gene flanking the enhanced green fluorescent protein (*EGFP*) gene was also designed (Figure S1D; Table S1). The gRNA and HDR template were cloned into the pMV-261-crRNA plasmid. Plasmids containing gRNA + HDR template, gRNA, or HDR template, as well as empty plasmid, were individually transformed into *M. tuberculosis*. A significant reduction in the number of colony-forming units (CFUs) was observed in the bacteria transferred with the plasmid carrying gRNA only when compared with bacteria transformed with the plasmid carrying gRNA + HDR template or the empty plasmid (Figure S1E). Sanger sequencing results of the CFUs transformed with the plasmids carrying gRNA + HDR template or gRNA only are shown in Figure S1F. The data suggested that self-DNA targeting causes a certain level of lethality to the bacteria and can be partially rescued using an HDR template. Given that the nonhomologous end joining (NHEJ) pathway of mycobacteria is efficient and actively participates in the DNA repair process [32], the rate of NHEJ contributing to the survival of the CFUs transformed with gRNA should be investigated.

### Reprogramming the endogenous type III-A CRISPR system for DNA editing

**Figure 2:**
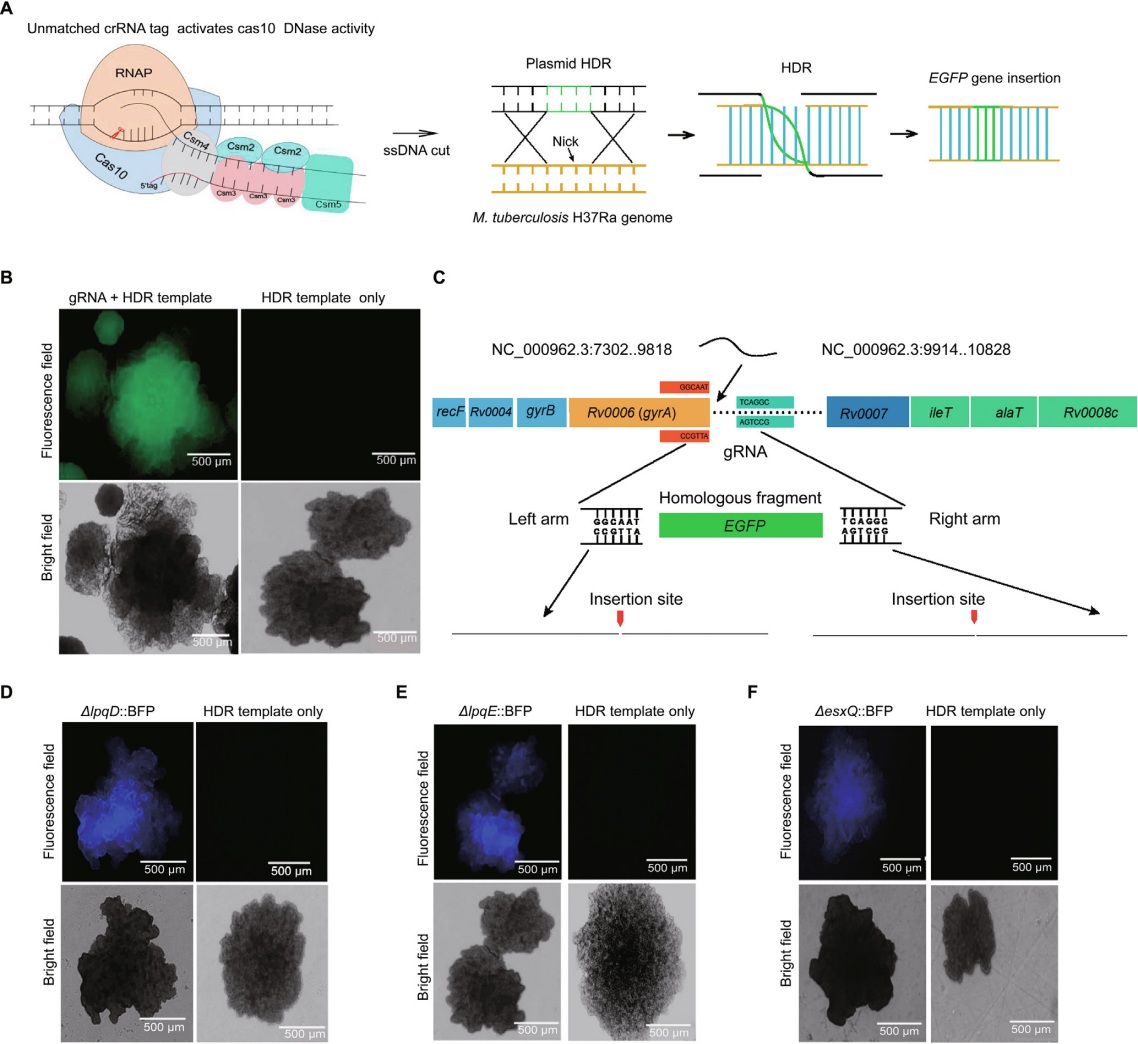
**CRISPR/Cas10-mediated gene knock-in and knockout in *M. tuberculosis*** **A.***M. tuberculosis* gene editing strategy in which CRISPR/Cas10 mediates specific DNA cleavage through a transcription-dependent process that can facilitate gene knock-in. **B.** Green fluorescence in bacteria with the *EGFP* gene integrated into the locus after *gyrA* by the endogenous CRISPR/Cas10 system. No fluorescence was detected in the control group transformed with the HDR template only. **C.** Schematic representation of *EGFP* insertion in the locus after *gyrA* and confirmation of insertion through Sanger sequencing. Fluorescence- and bright-field images of the colonies of *lpqE*-deleted (**D**), *lpqD*-deleted (**E**), and *esxQ*-deleted (**F**) strains. RNAP, RNA polymerase; ssDNA, single-stranded DNA; HDR, homology-directed repair; HR, homologous recombination.

Owing to the presence of an active endogenous type III-A CRISPR system in *M. tuberculosis*, we aimed to use this system to conduct gene knock-in in *M. tuberculosis*. To introduce an *EGFP* gene into the *M. tuberculosis* H37Ra genome to construct a strain expressing the GyrA-eGFP fusion protein, a 40-bp gRNA complementary to the coding strand of *gyrA* along with an HDR template containing an *EGFP* gene was inserted into pMV-261-crRNA and then transformed into *M. tuberculosis* (Figure 2A; Table S1). As shown in Figure 2B, fluorescence was observed in colonies transformed with the plasmid carrying gRNA + HDR template but not in colonies transformed with the plasmid carrying HDR template only. Upon examining approximately 100 colonies transformed with the plasmid carrying gRNA + HDR template, we observed fluorescence in more than 80% of the colonies. To confirm insertion of the *EGFP* gene in the correct position into the *M. tuberculosis* genome, fluorescent colonies were screened by PCR with the primer pair F and R (Figure S1D; Table S2). One band of 1.72 kb long was observed, indicating that the *EGFP* gene had been knocked in (Figure S2A). This finding was further confirmed by the Sanger sequencing of the PCR products (Figure 2C). These data demonstrated that the type III-A CRISPR system can be used for gene knock-in in *M. tuberculosis*.

Furthermore, we attempted to knock out nine genes belonging to the early secretory family (*esxA*, *esxB*, *esxQ*, *fbpB*, *espL*, and *hspX*) and the lipoprotein family (*lpqD*, *lpqE*, and *lpqN*) using the type III-A CRISPR system. For this purpose, 40-bp gRNAs targeting the coding strands of the corresponding genes (Table S1) were cloned into the pMV-261-crRNA plasmid individually. As a selection marker, the blue fluorescent protein (*BFP*) gene flanked by corresponding HDR template arms was cloned into plasmids containing gRNAs and then transformed into *M. tuberculosis*. Fluorescence was observed in colonies transformed with the plasmid containing gRNA + HDR template but not in the control colonies transformed with the plasmid containing the HDR template only (Figure 2D–F, Figure S2B). To further confirm the knockout of the target genes (*lpqD*, *lpqN*, *lpqE*, and *esxQ*), fluorescent colonies were selected for PCR amplification with specific primers (Table S2). The PCR products from *ΔlpqD*::BFP and *ΔlpqN*::BFP had the expected size (1.2 kb), indicating the replacement of respective genes by the *BFP* gene (Figure S2C and D). A 1.75-kb band indicating *ΔlpqE*::BFP was amplified from the colonies expressing BFP, and a 1.5-kb band was obtained after the amplification of the control colonies transformed with the plasmid containing HDR template only (Figure S2E). Similarly, the PCR amplification of the 1.6-kb *ΔesxQ*::BFP product verified the insertion of *BFP* at the desired locus (Figure S2F). The PCR products were further subjected to Sanger sequencing, which confirmed the replacement of target genes by the *BFP* gene (Figure S3).

### Efficiency and off-target analysis of type III-A CRISPR system-mediated DNA editing

**Figure 3:**
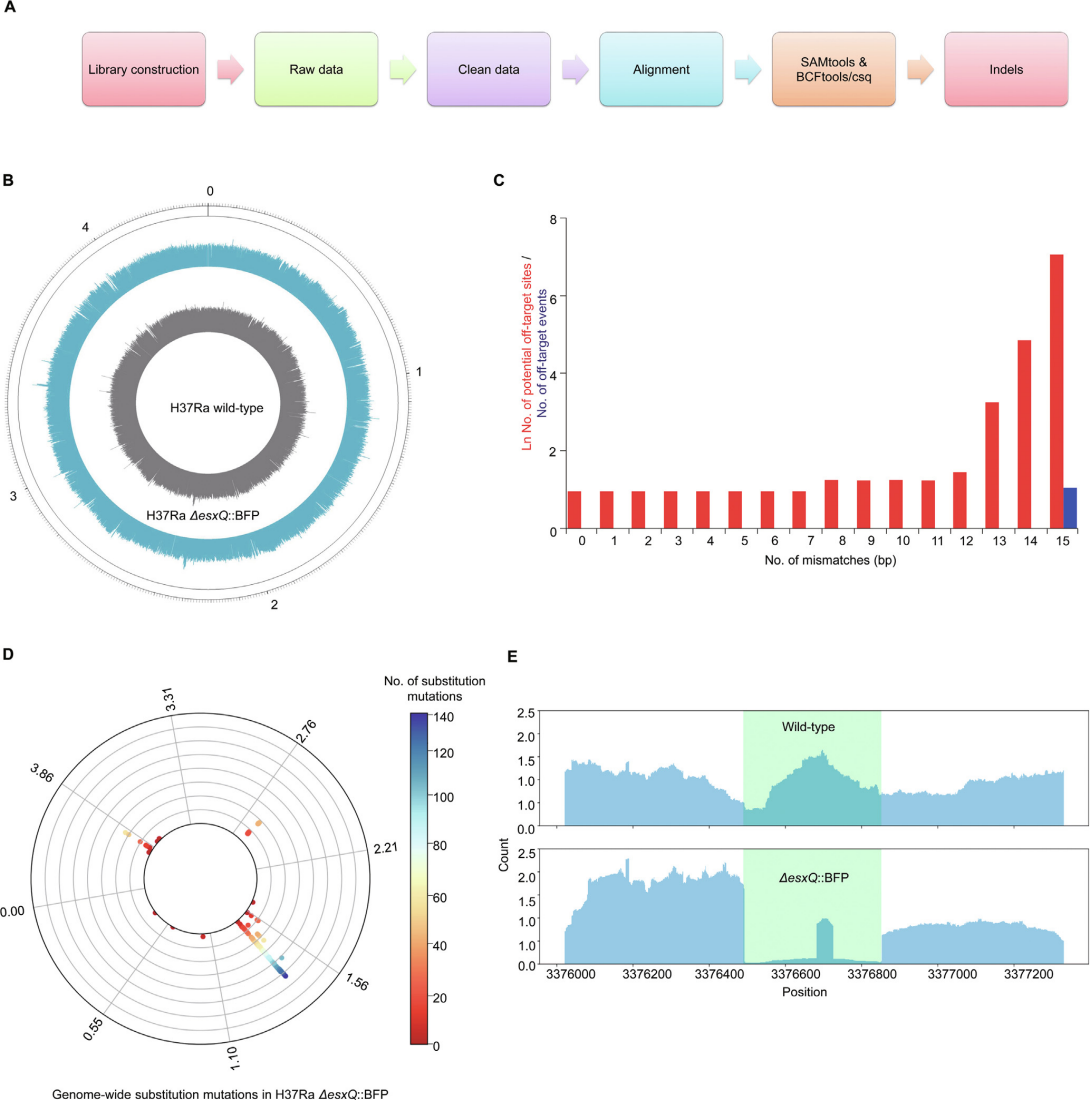
**Analyses of gene knockout efficiency and off-target effects** **A.** Schematic diagram for the gene knockout efficiency and off-target analyses from DNA library preparation to *in silico* analyses. **B.** Circos plot showing the genomic outline of the wild-type and *esxQ* knockout (*ΔesxQ*::BFP) mutant strains. **C.** Evaluation of whole-genome off-target effects in the *ΔesxQ*::BFP strain with different mismatch cutoff values. The 40-bp homologous fragments of a gRNA (with mismatches ≤ 15 bp) and their 20-bp upstream and downstream sequences are considered as potential off-target regions. No off-target effect was observed when mismatches ≤ 14 bp. **D.** Polar chart showing genome-wide substitution mutations in the *ΔesxQ*::BFP strain compared with the wild-type strain. The color indicates the number of substitution mutations at specific sites, and the circle represents the whole genome. **E.** Representation of the sequencing reads aligned on the *esxQ* locus in the wild-type and *ΔesxQ*::BFP strains.

To evaluate the off-target effects of the type III-A CRISPR system, the genomic DNA libraries of wild-type H37Ra, *ΔesxQ*::BFP, *ΔRv0839(esxC)*::BFP, and *ΔlpqD*::BFP strains were constructed and subjected to high-throughput sequencing (at least 250× for each sample; Figure 3A). The raw data were trimmed using Trimmomatic [33], and clean reads were mapped with the *M. tuberculosis* H37Ra reference genome using Burrows wheeler alignment (BWA) tool [34] (Figure 3B, Figure S4A and B). The off-target rate was examined with SAMtools [35] and BCFtools/csq [36]. The mutation events were analyzed in the potential gRNA off-target regions which contain the 40-bp homologous segements of a gRNA (with mismatches ≤ 15 bp) and their 20-bp upstream and downstream regions. No off-target mutations, including substitution and insertion–deletion (indel) mutations, were observed in the genome when mismatches ≤ 14 bp (Figure 3C and D). The statistical results showed the insertion of the *BFP* gene at the target position without any potential off-target indel mutations (Figure 3E, Figure S4C and D).

To check the genome editing efficiency of this system, we transformed *M. tuberculosis* H37Ra using a plasmid containing gRNA + HDR template corresponding to *lpqE* or a control plasmid containing HDR template only. Fluorescence was observed in approximately 80% of the colonies transformed with the plasmid containing gRNA + HDR template, whereas no fluorescence was observed in the colonies transformed with the control plasmid (Figure 2E). To verify the genome editing efficiency, 11 randomly picked colonies were cultured for 5–7 days and amplified with the primers of *lpqE*-KO-detection F and R (Table S2). *BFP* insertion was observed in 10 of the 11 clones, and only one clone showed the same band as that of the wild-type strain (Figure S4E). Sanger sequencing of these PCR bands showed that the *lpqE* gene was replaced by *BFP* (Figure S3C).

### Reprogramming the type III-A CRISPR system for RNAi in *M. tuberculosis*

**Figure 4:**
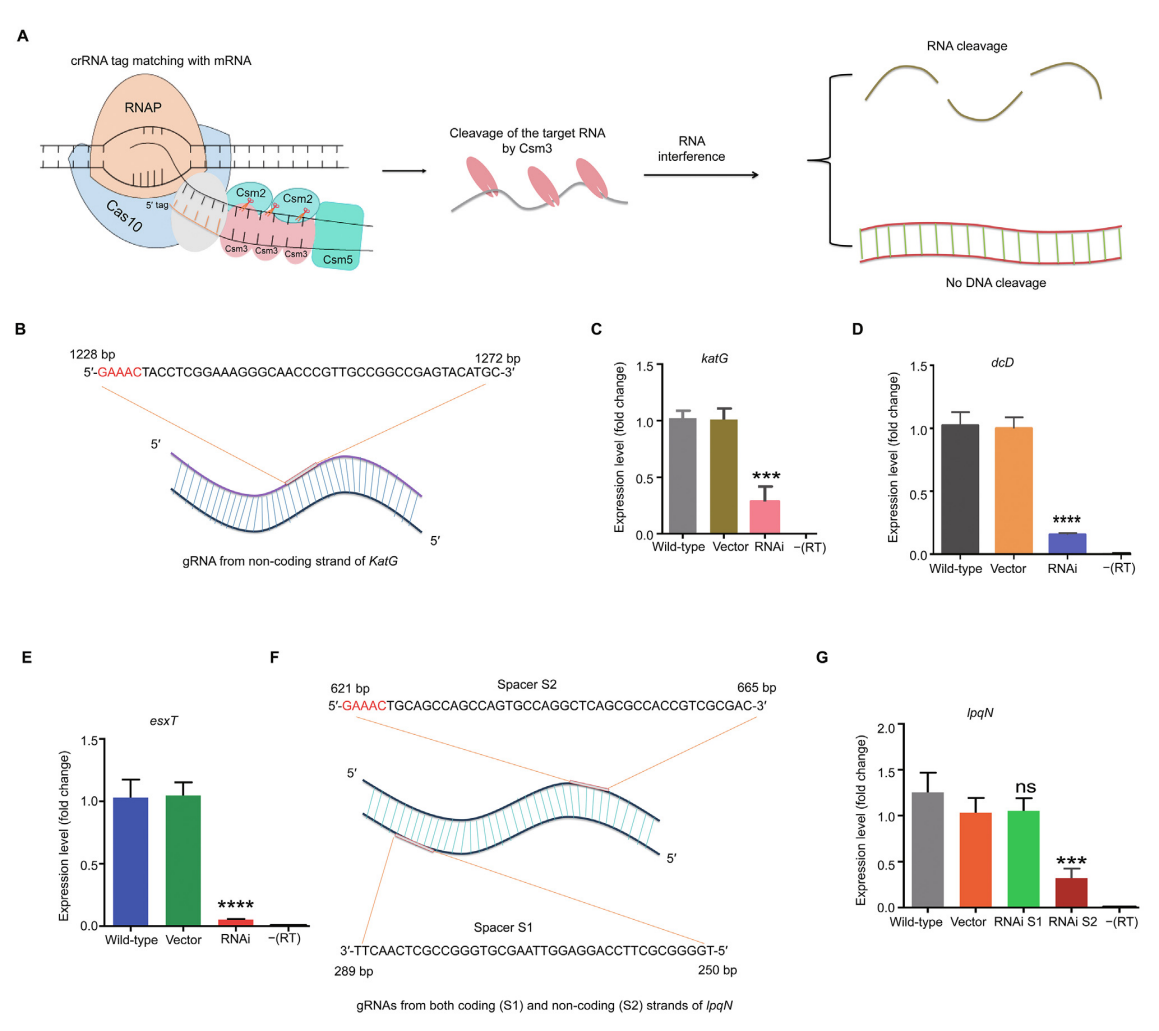
**CRISPR/Cas10-mediated single-gene interference in *M. tuberculosis*** **A.** Schematic for the CRISPR interference strategy in which the gRNA is flanked by a 5′-GAAAC-3′ tag at its 5′-end. This tag is recognized by the CRISPR/Cas10 complex, which stops the DNase activity of Cas10. The target RNA is cleaved by Csm3 without any DNA cleavage. **B.** Sequence and position of the gRNA designed for *katG* interference. The 40-bp region (1233–1272 bp) along with the pentanucleotide motif at 1228–1232 bp on the non-coding strand of *katG* was selected as the gRNA. **C.**–**E.** Inhibition of gene expression in the *katG* (C), *dcD* (D), and *esxT* (E) interference strains compared with the wild-type and control (empty vector) strains. **F.** Sequences and positions of the gRNAs designed for *lpqN* interference. **G.***lpqN* expression in the S1 gRNA and S2 gRNA strains compared with that in the wild-type and control (empty vector) stains. Student’s *t*-test (***, *P* < 0.001; ****, *P* < 0.0001; ns, not significant).

We aimed to reprogram the type III-A CRISPR system for gene silencing in *M. tuberculosis.* A 40-bp sequence flanked by a pentanucleotide motif 5′-GAAAC-3′ at its 5′-end (matching the repeat handle sequence) from the non-coding strand was selected as a gRNA. When the GAAAC motif of the crRNA completely matches with the 3′ anti-tag of the target RNA, this motif is recognized by Cas10 which stops the DNase activity of Cas10. The target RNA is then cleaved by Csm3 without DNA cleavage [37] (Figure 4A). Accordingly, a 40-bp sequence flanked by 5′-GAAAC-3′ at 1228–1232 bp from the non-coding strand of the *katG* gene was used as a gRNA for the specific targeting of the mRNA of the *katG* gene (Figure 4B; Table S1). The gRNA was cloned into pMV-261-crRNA and transformed into *M. tuberculosis*. qRT-PCR results showed that the expression of *katG* was significantly inhibited in the strain transformed with the plasmid carrying the gRNA compared with that in the wild-type and control (transformed with the empty plasmid) strains (Figure 4C). Then, two genes, *dcD* and *esxT*, were selected to investigate gene silencing with the same strategy. In this regard, a 40-bp sequence flanked by 5′-GAAAC-3′ from the non-coding strand of both genes was cloned into pMV-261-crRNA (Table S1). qRT-PCR results showed that the expression of both genes was significantly inhibited compared with the wild-type and control strains (Figure 4D and E).

To determine if gene silencing can be achieved by targeting the coding strand, we selected the *lpqN* gene as a target. Two gRNAs, designated as spacer S1 (targeting the coding strand) and spacer S2 (targeting the non-coding strand), were transformed into *M. tuberculosis*, respectively (Figure 4F). qRT-PCR results showed that the gRNA targeting the *lpqN* non-coding strand (spacer S2) efficiently inhibited the expression of *lpqN*, whereas the gRNA targeting the coding strand (spacer S1) did not inhibit gene expression (Figure 4G).

To check the polar effects of type III-A CRISPR system-mediated RNAi on the upstream and downstream regions of the target gene, we performed qRT-PCR on the neighbor genes of *lpqE* and *dcD* in the RNAi strains. The expression of genes flanking the *lpqE* gene was not significantly changed; however, the expression of genes flanking the *dcD* gene was significantly down-regulated relative to the wild-type strain (Figure S5A and B).

### Multiple-gene interference by the type III-A CRISPR system

**Figure 5:**
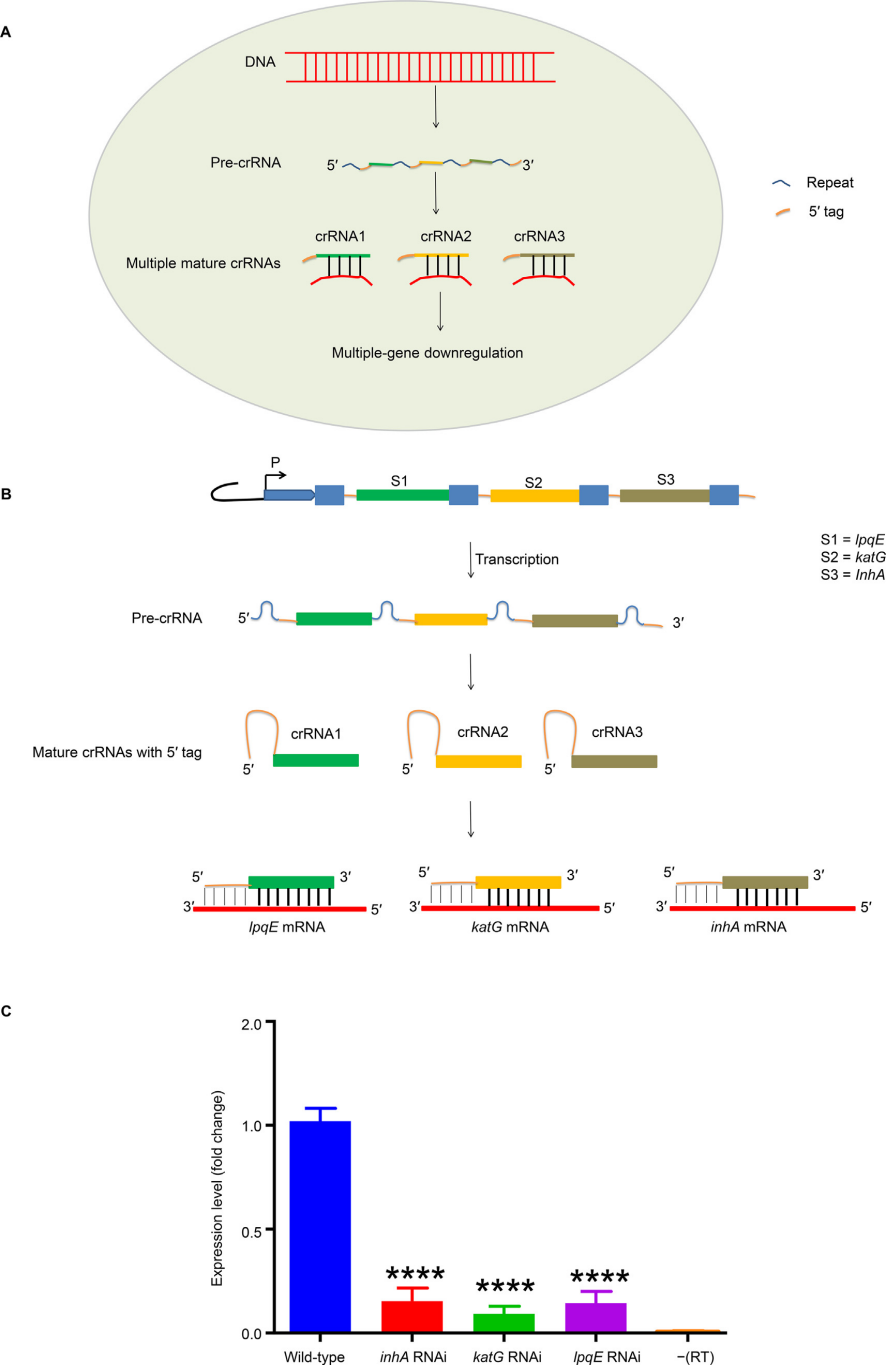
**CRISPR/Cas10-mediated multiple-gene interference in *M. tuberculosis*** **A.** Binding of multiple crRNAs to the corresponding targeted mRNAs. **B.** Schematic for the expression of multiple spacers, generation of multiple crRNAs, and binding of the crRNAs to the corresponding targeted mRNAs. **C.** Simultaneous gene expression inhibition of *lpqE*, *katG*, and *inhA* in the strain transformed with a plasmid containing the three spacers compared with the wild-type strain. Student’s *t*-test (****, *P* < 0.0001).

In the endogenous type III-A CRISPR system, the CRISPR array comprising multiple spacers flanked by repeats. This array is transcribed into pre-crRNA, which is then processed into mature crRNAs by endogenous Cas enzymes. The mature crRNAs bind to the corresponding sites on mRNAs, causing cleavage on multiple targeted genes (Figure 5A). Therefore, we aimed to perform multiple-gene silencing using the type III-A CRISPR as an interference system. Three spacers separately targeting *lpqE*, *katG*, and *inhA* flanked by repeats on both sides were synthesized and cloned into pMV-261-crRNA, and then transformed into *M. tuberculosis* (Figure 5B, Figure S5C). qRT-PCR results demonstrated that the expression of these three genes was simultaneously down-regulated with high efficiency compared with the wild-type strain (Figure 5C), indicating that the endogenous type III-A CRISPR system can be used for simultaneous silencing of multiple genes with high efficiency.

### Genome-wide RNAi screening in extracellular *M. tuberculosis*

**Figure 6:**
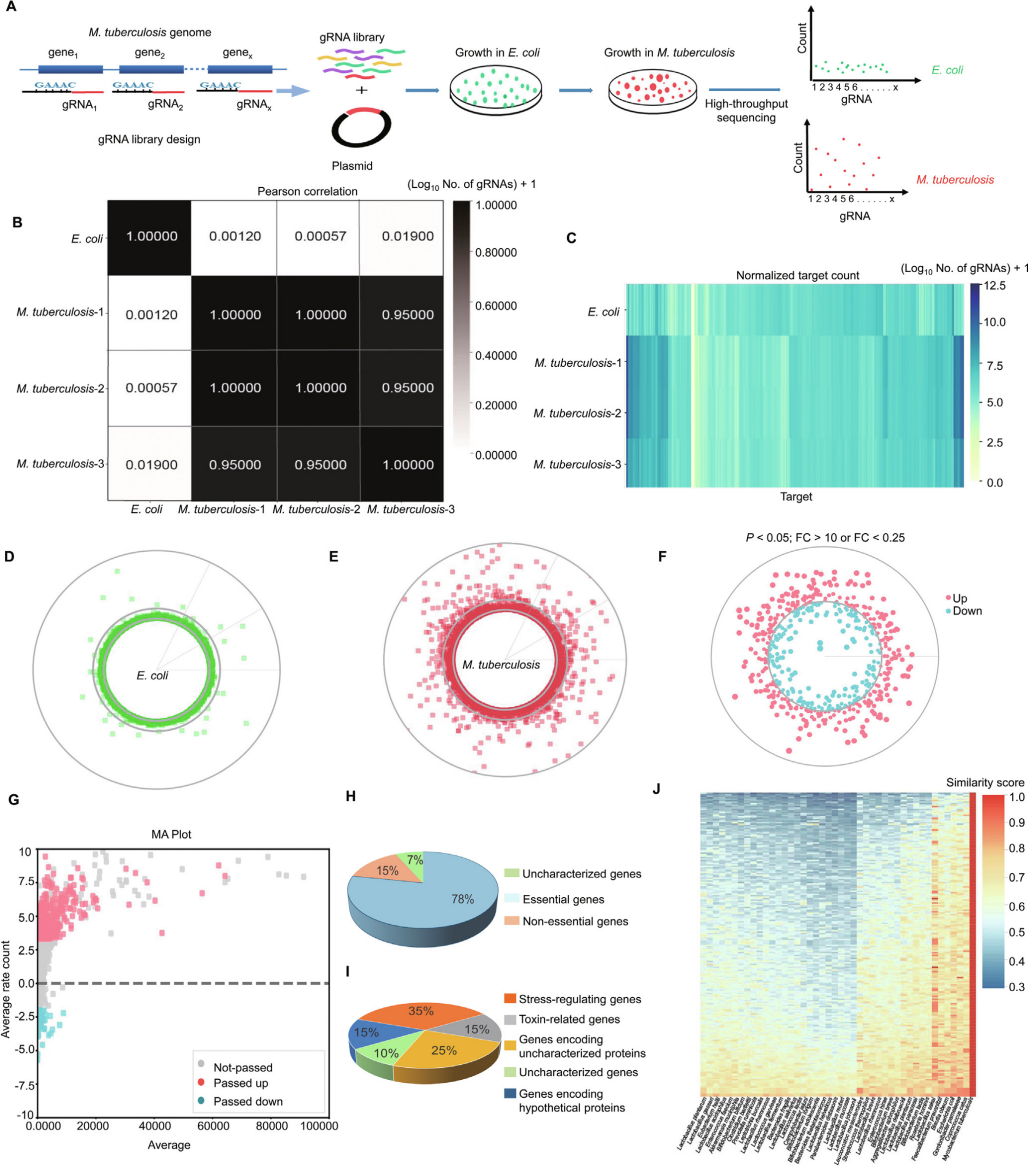
**CRISPR/Cas10-mediated genome-wide RNAi screening in extracellular *M. tuberculosis*** **A.** Schematic for gRNA library construction and genome-wide RNAi screening. **B.** Pearson correlation analysis of gRNA sequence count distributions of three independent *M. tuberculosis* samples with those of the *E. coli* (input) samples. **C.** Heatmap of the gRNA read counts of three independent *M. tuberculosis* samples compared with the *E. coli* inputs. **D.** and **E.** Global distribution of gRNA reads (indicated by mean value) in the *E. coli* genome (D) and *M. tuberculosis* genome (E). Each point represents a gRNA, and the radius represents the read count. **F.** Circle plot of the RNAi screening result. The red and blue points represent significantly up- and down-counted gRNAs (*M. tuberculosis vs. E. coli*), respectively, aligned to the corresponding target genes in the genome. The distance to the inner circle of each point represents the fold change value. **G.** MA plot of the RNAi screening result shown in (F). **H.** Pie chart showing the properties of the top 50 low-count target genes. **I.** Pie chart showing the properties of the top 50 high-count target genes. **J.** Heatmap showing the similarity of the 208 growth-facilitating genes between *M. tuberculosis* and the 43 most common probiotics.

We aimed to accomplish genome-wide RNAi screening with a reprogrammed type III-A CRISPR system. A library pool containing 5658 gRNA fragments was generated through on-chip oligo synthesis and cloned into the pMV-261-crRNA plasmid between *Xba*I and *Hind*III sites (Figure S6; Table S3). This library was first transformed into *E. coli* for plasmid amplification and gRNA input composition analysis. After amplification, this gRNA library was transformed into *M. tuberculosis* for the screening of growth-regulating genes (Figure 6A).

As a proof of concept, we collected approximately 1.5 million *E. coli* colonies and prepared a mixed gRNA construct library. This library was aliquoted and electro-transformed into three independent competent *M. tuberculosis* cells. After 3–4 weeks of incubation, approximately 1.5 million *M. tuberculosis* CFUs from 330 plates were harvested for plasmid preparation. The gRNAs were amplified from *E. coli* and *M. tuberculosis* plasmids through PCR and subjected to library construction for high-throughput sequencing.

We hypothesized that gRNAs with significantly reduced counts in *M. tuberculosis* compared with those in *E. coli* likely target growth-facilitating genes. By contrast, gRNAs with significantly increased counts probably target growth-repressing genes. We first screened the reads matched with the repeat-gRNA-repeat structure and then trimmed target sequences for further analysis. The counts of each target sequence were normalized using the median-of-ratios method [37]. The high Pearson correlation coefficients between the three independent *M. tuberculosis* gRNA libraries indicated that the composition and distribution of the target sequences and the corresponding read counts were highly similar; therefore, the screening assay is highly reproducible (Figure 6B). We analyzed the normalized count distribution of each target in the *E. coli* and *M. tuberculosis* libraries. The count distribution of most of the targets in the *E. coli* library was equally distributed, whereas the counts of many targets in the *M. tuberculosis* library were highly scattered (Figure 6C–E), suggesting that numerous gRNAs influence *M. tuberculosis* growth.

We further screened gRNAs with significantly reduced or increased counts in the *M. tuberculosis* library compared with the *E. coli* library (Figure 6F and G; Table S4). Finally, we identified 228 genes as potential *M. tuberculosis* growth-facilitating genes and 385 genes as potential *M. tuberculosis* growth-repressing genes (Table S4). Notably, among the top 50 potential growth-facilitating genes, 39 (78%) are essential for growth [38], [39], [40], [41], [42] (Figure 6H). This finding supports the reliability of the screening method. Among the top 50 potential growth-repressing genes, we found that 35% are stress-regulating genes, 15% are toxin-related genes, 15% are genes encoding hypothetical proteins, 10% are uncharacterized genes, and 25% are genes encoding uncharacterized proteins (Figure 6I).

Genes that facilitate growth or are essential for growth can be used as anti-TB drug targets. To further analyze for *M. tuberculosis* specific drug targets, we checked the similarity between the 208 growth-facilitating genes from *M. tuberculosis* and the whole genomes of 43 probiotics. The top 32 genes in the heatmap in Figure 6J revealed the least similarity score to the whole genomes of 43 probiotics. These data suggested that these genes are ideal anti-TB drug targets with conceivably fewer side effects on probiotics than other genes (Table S5).

### Genome-wide RNAi screening in intracellular *M. tuberculosis*

**Figure 7:**
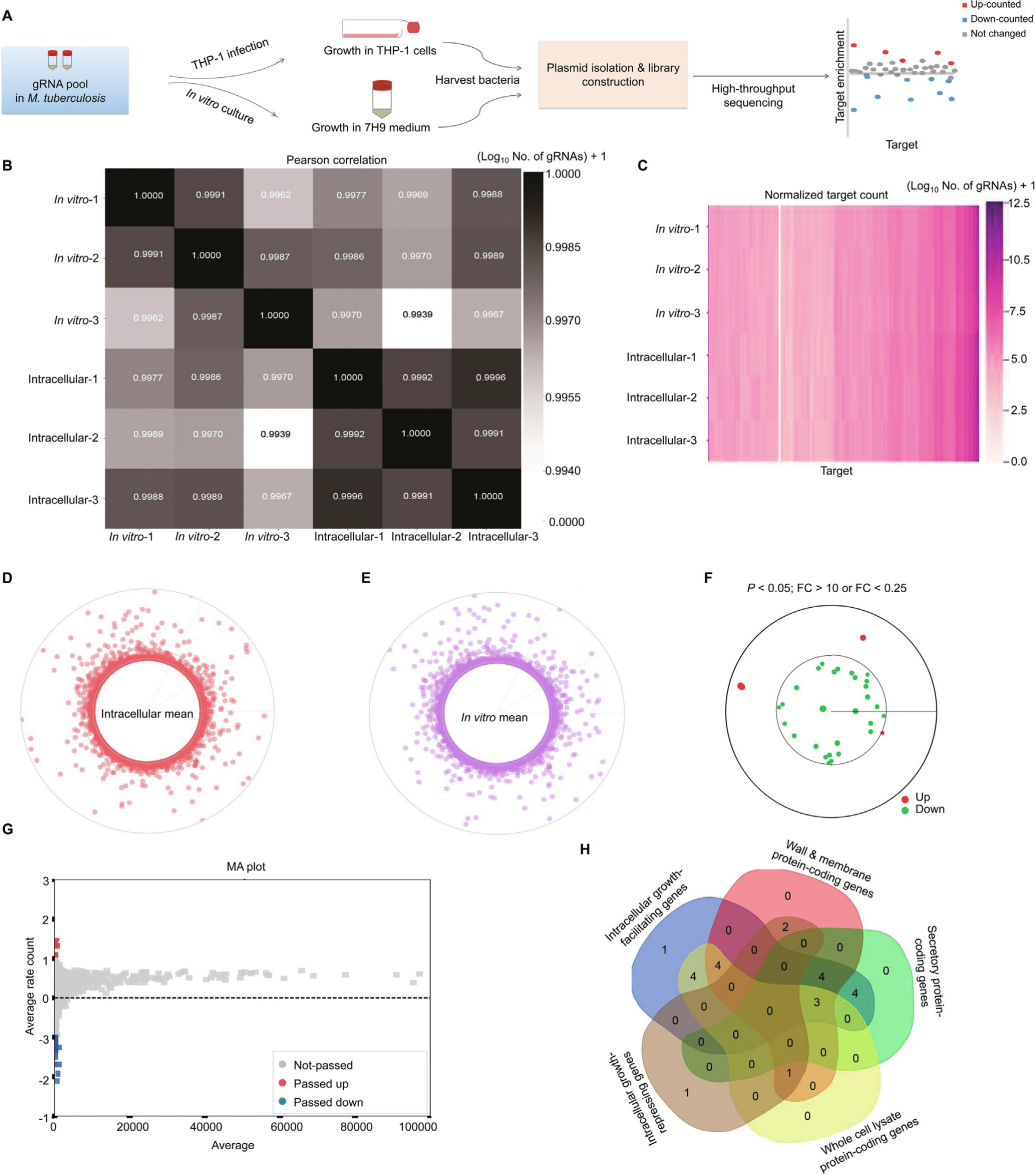
**CRISPR/Cas10-mediated genome-wide RNAi screening in intracellular *M. tuberculosis*** **A.** Schematic for CRISPR/Cas10-mediated genome-wide RNAi screening for *M. tuberculosis* intracellular growth-regulating genes. **B.** Pearson correlation analysis of gRNA sequence count distributions of three independent intracellular *M. tuberculosis* samples compared with three independent *in vitro* cultured *M. tuberculosis* samples. **C.** Heatmap of the gRNA read counts of three independent intracellular *M. tuberculosis* samples compared with three independent *in vitro* cultured *M. tuberculosis* samples. **D.** and **E.** Global distribution of gRNA reads (indicated by mean value) in intracellular (D) and *in vitro* cultured (E) *M. tuberculosis*. Each point represents a gRNA, and the radius represents the read count. **F.** Circle plot of the RNAi screening result. The red and green dots represent significantly up- and down-counted gRNAs (intracellular *vs. in vitro*), respectively, aligned to the corresponding target genes in the genome. The distance to the inner circle of each point represents the fold change value. **G.** MA plot of the RNAi screening result is shown in (F). **H.** Venn diagram showing the different categories of 24 intracellular growth-regulating genes.

Finally, we applied this system to perform genome-wide RNAi screening on growth-regulating genes during intracellular growth (*i.e.*, growth inside macrophages). Approximately 1.5 million *M. tuberculosis* CFUs containing a library of 5658 gRNAs in triplicate were used to infect THP-1 cells and grown for 3 days after the removal of extracellular bacteria. As a reference, *M. tuberculosis* CFUs containing 5658 gRNAs in triplicate were cultured *in vitro* in Middlebrook 7H9 broth (Figure 7A). In this assay, gRNAs with significantly reduced counts during intracellular growth likely target the genes highly required for intracellular *M. tuberculosis* growth (Figure 7A).

The gRNAs from the *in vitro* cultured and intracellular bacteria were amplified and subjected to library construction for high-throughput sequencing. The raw reads were screened according to the repeat-gRNA-repeat structure and then normalized using the median-of-ratios method [40]. As shown in Figure 7B, the high Pearson correlation coefficients between the three independent gRNA libraries from either *in vitro* cultured or intracellular bacteria indicated that the composition and distribution of the target sequences and the corresponding read counts are highly reproducible. We analyzed the normalized count distribution of each gRNA target in the *in vitro* and intracellular groups (Figure 7C–E) and screened gRNAs that were significantly reduced or increased in number in the intracellular groups compared with the *in vitro* groups (Figure 7F and G). Finally, we identified 29 low-count genes as potential *M. tuberculosis* intracellular growth-facilitating genes and 4 high-count genes as potential intracellular growth-repressing genes (Table S6). Among the top 24 highly growth essential genes, 11 were secretory protein-coding genes, 14 were involved in the coding of membrane and cell wall component proteins, and 12 genes code for the whole cell lysate proteins [43], [44], [45] (Figure 7H).

## Discussion

In the present study, we reprogrammed an endogenous type III-A CRISPR system of *M. tuberculosis* to develop a robust and versatile toolbox for gene knock-in/knockout process, gene silencing, and whole-genome RNAi screening. In addition to its high efficiency, this system has the advantage of a simple procedure, requiring only two-step cloning (a gRNA and its cognate donor DNA). By contrast, phage transduction for gene editing is laborious and time-consuming because of the involvement of multiple cloning steps and the screening for positive phages. This tool requires expensive kits and expertise [5], [46].

To the best of our knowledge, the present study is the first to demonstrate the use of type III-A CRISPR system-mediated “killing two birds with one stone” tool in robust gene editing and silencing in *M. tuberculosis*. The endogenous type III-A CRISPR-based specific gene silencing system was obtained by simply transforming a plasmid expressing a mini-CRISPR array carrying a gRNA into *M. tuberculosis*.

After transcription, the mini-CRISPR array harnesses endogenous Csm complexes to target genes/transcripts, thus inhibiting the expression of target genes. This system has several advantages over the CRISPR/Cas9 system. It is a simple system for cloning a mini-CRISPR array carrying a gRNA and does not require the introduction of any exogenous *cas9* gene, thus minimizing the toxicity of the system. In contrast to Cas9, the stringent requirement of the PAM sequence for gene targeting is not required. Similar to the endogenous type III-A CRISPR system, the reprogrammed CRISPR array can simultaneously generate multiple spacers. These spacers can be processed into multiple mature crRNAs that bind to corresponding target sites and inhibit the expression of multiple genes simultaneously. This system can solve the problem of functional redundancy in functional genomic research on *M. tuberculosis*.

*M. tuberculosis* causes the leading infectious disease in the world, and the pathophysiology and functional genomics of this organism remain poorly understood. Thus, the development of novel anti-TB drugs and vaccines is hampered*.* Whole-genome screening through gene silencing based on a CRISPR/Cas system is a powerful approach and has been widely used for exploring gene functions [39], [47]. Wet et al. [48] applied CRISPR-dCas9 screening for the functional characterization of transcription factors in *M. smegmatis*. However, no study on *M. tuberculosis* has been performed on the genome scale. In this study, we designed 5656 and 5658 gRNAs for the H37Rv and H37Ra strains, respectively, covering the majority of the coding sequences of the *M. tuberculosis* genome. We used the gRNA library designed for H37Ra for a whole-genome-scale screening of growth-regulating genes as a proof of concept. Using this approach, we identified 228 potential growth-facilitating genes and 385 potential growth-repressing genes from H37Ra.

Notably, among the top 50 potential growth-facilitating genes, 78% are essential for growth. This finding supports the reliability of the screening system. Given that most of the RNAi-identified growth-facilitating genes are essential for bacterial growth, these genes can be used as anti-TB drug targets. In this study, we further analyzed the similarity between the growth-facilitating genes of *M. tuberculosis* and the whole genomes of 43 probiotics and identified 32 genes coding for potential anti-TB drug targets that possibly have fewer side effects on probiotics than the other identified genes. The identification of novel drug targets can be of great importance to protein structure-based drug design and screening, given the current severity of antibiotic resistance in *M. tuberculosis*.

RNAi screening identified a certain number of toxin- and stress-related genes as potential growth-repressing genes. Interference of the toxin-related genes can promote the growth of *M. tuberculosis* through the attenuated repression of toxin proteins. Therefore, our database containing growth-repressing genes may be used for screening toxin–antitoxin family genes. In this regard, previously identified antitoxin genes, such as *dinX* and *vapB5*, were present in our database [49]*.* Furthermore, the knockdown of some stress regulons and other transcriptional factor regulators for non-replicating persistence, such as *devR/dosR* and *sigA,* can promote the growth of *M. tuberculosis* [50]. Thus, our screening approach may contribute to the identification of genes involved in toxin–antitoxin regulation, stress regulation, and transition from active *M. tuberculosis* to its persistent form. The procedure for this screening system requires only one step of gRNA library cloning and electro-transformation into *M. tuberculosis* and is much simpler and more cost-effective than other screening systems, such as transposon-based mutagenesis. Moreover, in contrast to gene knockout, this screening system is based on gene expression inhibition, thus allowing genetic screening and functional investigation of the genes essential for growth.

In our study, we screened the genes regulating growth in host cells. We identified 29 down-regulated genes as potential intracellular growth-facilitating genes and four up-regulated genes as potential intracellular growth-repressing genes for *M. tuberculosis*. Among the top 20 highly down-regulated genes, 18 genes are essential for growth inside macrophages [51], [52], [53], [54], [55], [56], [57], while the 2 other genes were novel *M. tuberculosis* intracellular growth-facilitating genes (inside THP-1 derived macrophages). Of the highly down-regulated genes, s*enX3* belongs to the two-component regulatory system and *groEL1* encodes a membrane protein of *M. tuberculosis*. Both genes are required for the survival of *M. tuberculosis* inside the macrophages but not *in vitro* [58], [59]. As these genes are dispensable for the *in vitro* growth of *M. tuberculosis* but essential during intracellular survival, they are potential vaccine candidates for TB. Among the intracellular growth-repressing genes, *ponA-*1 is involved in stress regulation [60], [61]. Determining whether the inhibition of this gene can prevent *M. tuberculosis* from going to a latency stage inside macrophages is of great importance.

The limitation of this endogenous CRISPR system-based gene silencing approach is its dependency on the pentanucleotide motif (GAAAC) for gene silencing, though the system can cover a majority of the *M. tuberculosis* genome.

In summary, we reprogrammed an *M. tuberculosis* endogenous type III-A CRISPR/Cas10 system for simple and efficient genome editing, gene interference, and genome-wide RNAi screening. This system greatly facilitates genetic manipulation with high specificity and efficiency. This system can be extensively used for exploring the functional genomics of *M. tuberculosis* and will contribute to basic research on *M. tuberculosis* biology and the development of anti-TB drugs and vaccines.

## Materials and methods

### Bacterial culturing and transformation

*E. coli* was cultured in a lysogeny broth (LB) medium (with or without kanamycin) for 12–14 h at 37 °C in a shaking incubator. *M. tuberculosis* H37Ra was cultured in Middlebrook 7H9 broth (Catalog No. 271310, BD Biosiences, Radnor, PA) supplemented with 10% oleic albumin dextrose catalase (OADC; Catalog No. M0678-1VL, Sigma-Aldrich, Darmstadt, Germany), 0.02% Tween-80, and 0.5% glycerol or on Middlebrook 7H10 agar (Catalog No. 262710, BD Biosiences) petri plate supplemented with 10% OADC and 0.5% glycerol [with or without kanamycin (25 μg/ml)]. For *in vitro* growth analysis, the bacteria were cultured in Middlebrook 7H9 broth, and OD_600 nm_ was adjusted to 0.15. The bacterial suspension (0.2 ml) was used for examining OD_600 nm_ every day for 18 days. All experiments were performed in triplicate for each sample.

For preparation of the competent cells of *M. tuberculosis*, 100 ml of *M. tuberculosis* H37Ra were pelleted through centrifugation at 8000 *g* for 10 min at 4 °C and washed three times with 10% ice-chilled glycerol. The pellet was then suspended with 4 ml of 10% ice-chilled glycerol and aliquoted into Eppendorf tubes with each containing 200 μl of suspension. For electro-transformation, plasmids of interest were electro-transformed into the competent cells of *M. tuberculosis* H37Ra using Gene Pulser Xcell Total System (Catalog No. 1652660, BIO-RAD, Hercules, CA) with default settings (2.5 kV, 25 µF, and 1000 Ω resistance). The bacterial suspension was transferred to a fresh Eppendorf tube containing 1 ml of Middlebrook 7H9 broth. The cell suspension was pelleted after 24 h of incubation and plated onto Middlebrook 7H10 agar plates containing kanamycin.

### Cell culture and infection

Human monocyte cell line THP-1 (ATCC TIB-202) was cultured in RPMI-1640 complete growth medium supplemented with 10% FBS and then differentiated for 24 h using a culture medium containing 40 ng/ml phorbol 12-myristate 13-acetate (PMA) before infection. For infection, the cells were seeded into a T75 flask for 24 h and incubated with H37Ra (multiplicity of infection = 10) for 6 h at 37 °C in 5% CO_2_. The cells were washed three times with prewarmed PBS to remove extracellular bacilli and supplied with fresh RPMI-1640 complete growth medium containing amikacin (50 μg/ml). The intracellular bacteria were isolated 3 days post infection and subjected to plasmid isolation.

### Plasmid construction and preparation

For pMV-261-crRNA plasmid construction, two 36-bp repeats from *M. tuberculosis* H37Ra type III-A CRISPR array containing two *Bbs*I sites in between were commercially synthesized (Wuhan GeneCreate Biological Engineering, Wuhan, China) and cloned into pMV-261. A 40-bp gRNA sequence corresponding to the sequence of the spacer in the CRISPR array was selected from the target genes. The spacer fragments were generated by annealing the corresponding complementary oligonucleotides and cloned into the pMV-261-crRNA plasmid at the *Bbs*I sites to generate pMV-261-gRNA. The gRNA sequences corresponding to each gene are listed in Table S1. HDR templates containing either *EGFP* or *BFP* flanked by 400-bp left and right arms of the corresponding target gene were generated by overlapping PCR using specific primers (Table S2), and inserted into pMV-261-crRNA at the *Pst*I site. For the gene knockout strategy, the HDR templates were generated to replace the whole coding sequence of the target gene with *BFP*. For single-gene silencing, a 40-bp sequence as well as its 5′-end pentanucleotide motif (5′-GAAAC-3′) from the non-coding strand of the target gene was taken as the gRNA. The gRNA sequences of the target genes are listed in Table S1. For multiple-gene silencing, the plasmid containing three 45-bp spacers each flanked by 36-bp repeats on both sides were commercially synthesized (Wuhan GeneCreate Biological Engineering). The sequences are listed in Table S1.

### Fluorescence imaging

For identification of transformants, the fluorescent colonies were either directly observed or re-suspended in sterilized phosphate buffer saline and spread on the microscopic slide and then examined using Olympus IX73 microscope (Japan). Each selected colony was imaged using a filter with the excitation/emission wavelengths of 465–495 nm/515–555 nm for GFP and 55–375 nm/400 nm for BFP. All experiments were performed in triplicate.

### Identification of gene knock-in/knockout

Specific primers designed in the upstream and downstream regions of the HDR template (F and R) or in the fluorescent gene (R1) used for PCR screening of mutant strains are listed in Table S2. The PCR product was sequenced by Sanger sequencing (TsingKe Biological Technology, Wuhan, China) and analyzed by NCBI BLAST.

### RNA extraction and quantitative real-time PCR

*M. tuberculosis* H37Ra was pelleted and re-suspended in 1 ml Trizol reagent (Catalog No. 15596-026, Takara, Shiga, Japan) and then homogenized using lysing matrix B (Catalog No. D1031-01, MP Bio Medical, Kaysersberg, France). Next, 0.2 ml of chloroform was added to the bacterial suspension and centrifuged at 10,000 r/min for 10 min at 4 °C, and then the aqueous layer was transferred to a new RNase-free Eppendorf tube. Isopropanol was used to precipitate RNA at room temperature for 10 min. RNA was then pelleted and washed twice with freshly made 80% ethanol. The RNA pellet was air-dried, dissolved in DEPC water, and treated with TURBO DNase (Catalog No. AM2238, Ambion, Austin, TX) to remove the genomic DNA contamination. RNA was reverse transcribed to cDNA using ReverTra Ace qPCR RT Kit (Catalog No. FSQ-101, Toyobo, Shiga, Japan). qRT-PCR was carried out using PerfeCTa SYBR Green SuperMix (Catalog No. 66186691, Quanta BioSystem, Osaka, Japan) and Applied Biosystems 7300 real-time PCR system with Sequence Detection Software (v1.4.0). Data were analyzed using the 2^−ΔΔCT^ method, and *gyrB* was used as the reference gene. The primers used for qRT-PCR are listed in Table S2.

### gRNA library designing and construction

The 40-bp sequence with the ‘GAAAC’ motif at its 5′-end in the *M. tuberculosis* H37Ra genome sequence (Genome assembly: ASM1614v1) was selected as the gRNA candidate sequence in the *M. tuberculosis* H37Ra genome (Figure 7A, Figure S6; Table S3). The library pool containing 5658 target fragments with identical ends was generated through on-chip oligo synthesis (Twist Biosciences, San Francisco, CA) (Table S3). The gRNA pool was dissolved in nuclease-free TE buffer (pH 8.5) to a final concentration of 2.5 ng/ml and then amplified with Phanta Max Super-Fidelity DNA Polymerase Kit (Catalog No. P505, Vazyme, Nanjing, China) for 12 cycles. The Library-Seq primers F and R (Table S2) were used, and amplified products were run on 2% agarose gel and purified with an OMEGA Gel Extraction Kit (Catalog No. D2500-01, OMEGA Bio-Tek, Norcross, GA). The library was cloned into pMV-261-crRNA through homolog recombination using ClonExpress II One Step Cloning Kit (Catalog No. C112, Vazyme). The recombinant plasmids were then transformed to highly competent *E. coli* DH5α. The efficiency and construction of the library were validated through Sanger sequencing.

### Library screening and high-throughput sequencing

For genome-wide RNAi screening, plasmid DNA was isolated from all three cultures of *M. tuberculosis* H37Ra. Then, 20 µl of the plasmids from each sample were used as template to amplify the repeat-gRNA-repeat structure (Figure S4). Approximately 20 µl of the plasmids isolated from *E. coli* were used as a reference. Briefly, 20 µl of the plasmids of each sample were amplified with Library-Seq primers F and R using Phanta Max Super-Fidelity DNA Polymerase Kit (Catalog No. P505, Vazyme). The PCR reaction was set as follows: 98 °C for 4 min, 20 cycles of 98 °C for 10 s, 56 °C for 10 s, and 72 °C for 25 s, followed by 72 °C for 5 min and 4 °C hold. The sequencing library of the gRNA library was prepared using VAHTS Universal DNA Library Prep Kit V3 for Illumina (Catalog No. ND607-02, Vazyme) according to the manufacturer’s instruction.

The fragments were treated with End Prep Mix for end repairing, 5′ phosphorylation, dA-tailing, and purification. AMPure XP beads (Catalog No. A63881, Beckman, Indianapolis, IN) were used. The fragments were ligated using indexed adapters with “T” overhangs. The ligated products were purified using the VAHTS DNA clean beads (Catalog No. N411-10-AA, Vazyme) and amplified through PCR for 10 cycles. The libraries were run on a gel and purified, and then validated with an Agilent 2100 bioanalyzer (Agilent Technologies, Santa Clara, CA). For *M. tuberculosis* H37Ra whole-genome sequencing, whole genomic DNA was isolated and subjected to fragmentation through sonication with Diagenode Bioruptor Pico system (Diagenode, Denville, NJ). The fragmented DNA was run on 1% agarose gel, and the 150–350-bp fragments were purified, end-repaired, ligated with adapters, and sequenced after amplification. Then, the libraries with different indexes were pooled and loaded on Illumina HiSeq-X-Ten System for sequencing (Illumina, San Diego, CA).

### High-throughput sequencing data analysis

For *M. tuberculosis* whole-genome sequencing analysis, the adapters were trimmed from raw data with Trimmomatic [33]. Clean data for each accession were mapped to the *M. tuberculosis* H37Ra reference genome (Genome assembly: ASM76770v1) with BWA [34] at default settings. Substitution and indel detection was achieved using SAMtools and BCFtools/csq [35], [36], [62]. EDILIB was used [63] to align a gRNA against the reference genome with up to 15 mismatches, which were visualized as a bar plot. The 40-bp homologous segments of the gRNA (with mismatches ≤ 15) and their 20-bp upstream and downstream regions were selected as potential off-target regions [64]. Bedtools (v2.26.0) was used to validate gene knockout [65].

For RNAi analysis, raw data were filtered according to the repeat-gRNA-repeat structure. Then, the gRNA sequences were extracted and counted. To normalize the total reads from different libraries to the equivalent size and maintain the read composition of each gRNA, we normalized the total count of each sample with the following formula:

$$NTSC_{ij}=\frac{TSC_{ij}}{s_{j}}$$

where $TSC_{ij}$ represents the i^th^ gRNA count in sample j and is the size factor of sample j. The median-of-ratios method in the DESeq R package was applied to TSC normalization [66]: $s_{j}={\mathrm{median}}_{\text{i}}\frac{TSC_{ij}}{{\left(\prod_{v=1}^{m}TSC_{iv}\right)}^{1/m}}$.

After normalization, the read count ratio of each target between *M. tuberculosis* and *E. coli* was calculated. For the screening of significantly increased/decreased targets, *P* value and fold change were considered. *P* value was calculated by independent *t*-test and then adjusted with the Benjamini-Hochberg method. The *P* value threshold was 0.05. The fold change threshold was 0.25 for down-counted targets and 10 for up-counted targets.

To check the similarity of significantly down-counted *M. tuberculosis* genes with the whole genomes of the 43 most common probiotics (https://www.kegg.jp/kegg/catalog/org_list.html), the classic Needleman-Wunsch algorithm was applied [67]. The similarity was presented as a heatmap, which was generated using the heatmap R package (https://cran.r-project.org/web/packages/pheatmap/).

## Data availability

The *M. tuberculosis* genome sequence data reported in this study have been deposited in the Genome Sequence Archive [68] at the National Genomics Data Center, Beijing Institute of Genomics, Chinese Academy of Sciences / China National Center for Bioinformation (GSA: CRA003057), and publicly available at https://ngdc.cncb.ac.cn/gsa.

## CRediT author statement

**Khaista Rahman:** Conceptualization, Investigation, Software, Data curation, Methodology, Writing - review & editing. **Muhammad Jamal:** Conceptualization, Software, Data curation, Methodology, Validation, Writing - original draft. **Xi Chen:** Visualization, Writing - review & editing, Validation. **Wei Zhou:** Software, Methodology, Writing - review & editing. **Bin Yang:** Visualization, Writing - review & editing. **Yanyan Zou:** Software, Data curation, Formal analysis, Writing - review & editing. **Weize Xu:** Formal analysis, Writing - review & editing, Methodology. **Yingying Lei:** Visualization, Writing - review & editing, Validation. **Chengchao Wu:** Formal analysis, Writing - review & editing. **Xiaojian Cao:** Writing - review & editing. **Rohit Tyagi:** Writing - review & editing. **Muhammad Ahsan Naeem:** Writing - review & editing. **Da Lin:** Writing -review & editing, Visualization. **Zeshan Habib:** Writing - review & editing. **Nan Peng:** Writing - review & editing, Resources. **Zhen F. Fu:** Writing - review & editing, Resources. **Gang Cao:** Conceptualization, Supervision, Resources, Project administration, Writing - review & editing, Funding acquisition. All authors have read and approved the final manuscript.

## Competing interests

The authors declare no conflict of interest.

## Supplementary material

Supplementary Figure S1 **Functional analysis of M. tuberculosis type III-A CRISPRS system A.** Vector map of the construct for CRISPR-mediated gene editing and interference. **B.** Sanger sequencing chromatographs of qRT-PCR products of the respective *cas/csm* genes. **C.** Sequence information of *Psmyc* promoter, gRNA, repeats, and terminator. **D.** Cartoon representation of a general HDR template used for gene insertion and deletion. The black arrows with R and F labels on both ends of the HDR template represent the positions of the primers that could be used to confirm the insertion or deletion. **E.** Self-DNA is targeted by the endogenous type III-A CRISPR system directed by a gRNA designed for the *gyrA* gene. The graph represents the number of CFUs of *M. tuberculosis* transformed with gRNA, HDR, gRNA plus HHDR or empty plasmid. F. Sanger sequencing of the CFUs transformed with the HDR template and gRNA and those transformed with gRNA only.

Supplementary Figure S2 **Type III-A CRISPR system-mediated gene editing in *M. tuberculosis*. A.** PCR confirmation of *EGFP* knock-in into the gyrA locus. **B.** Blue fluorescence in bacterial colonies expressing *BFP* after replacement of the target gene. **C.–F.** PCR confirmation of gene knockout.

Supplementary Figure S3 Representative Sanger sequencing chromatographs revealing *BFP* insertion into the target sites**.**

Supplementary Figure S4 **Efficiency and off-target evaluation of type III-A CRISPR-mediated gene deletion A.** and **B.** Circos plots showing the genomic outlines of the *ΔesxC*::BFP and *ΔlpqD*::BFP strains along with their wild-type strains. **C.** and **D.** Representation of the sequencing reads aligned on the *esxC* and *lpqD* locus in the wild-type and respective mutant strains. **E.** PCR amplification showing successful insertion of *BFP* in 10 out of 11 colonies. The bands on the gel from 1 to 11 represent H37Ra transformed with the plasmid containing the gRNA plus HDR template corresponding to *lpqE* gene.

Supplementary Figure S5 **Type III-A CRISPR-mediated RNAi in *M. tuberculosis* A.** and **B.** Polar effects of the endogenous type III-A CRISPR-mediated RNAi on the neighbor genes of *lpqE* and dcD. **C.** Sequences of the spacers used for multiple-gene interference.

Supplementary Figure S6 **Strategy for gRNA library design for genome-wide RNAi screening in *M. tuberculosis***

Supplementary Table S1 **List of gRNA sequences used for gene knockout/knock-in and RNA interference**

Supplementary Table S2 **List of primers for gene knockout, knock-in, knockout, and qRT-PCR analysis**

Supplementary Table S3 **List of gRNA sequences synthesized for genome-wide RNAi screening**

Supplementary Table S4 **List of deferentially distributed gRNA sequences obtained during genome-wide RNAi screening for in vitro growth-regulating genes of *M. tuberculosis***

Supplementary Table S5 **List of *M. tuberculosis* drug target genes based on in vitro screening method**

Supplementary Table S6 **List of deferentially distributed gRNA sequences obtained during genome-wide RNAi screening for intracellular growth-regulating genes of M. tuberculosis during infecting macrophages**
