# Supplementary figures and images for: Reprogramming Mycobacterium tuberculosis CRISPR System for Gene Editing and Genome-wide RNA Interference Screening
Source: Genomics Proteomics Bioinformatics. 2021 Dec 16;20(6):1180–96. doi: 10.1016/j.gpb.2021.01.008 (PMC10225669; doi:10.1016/j.gpb.2021.01.008)

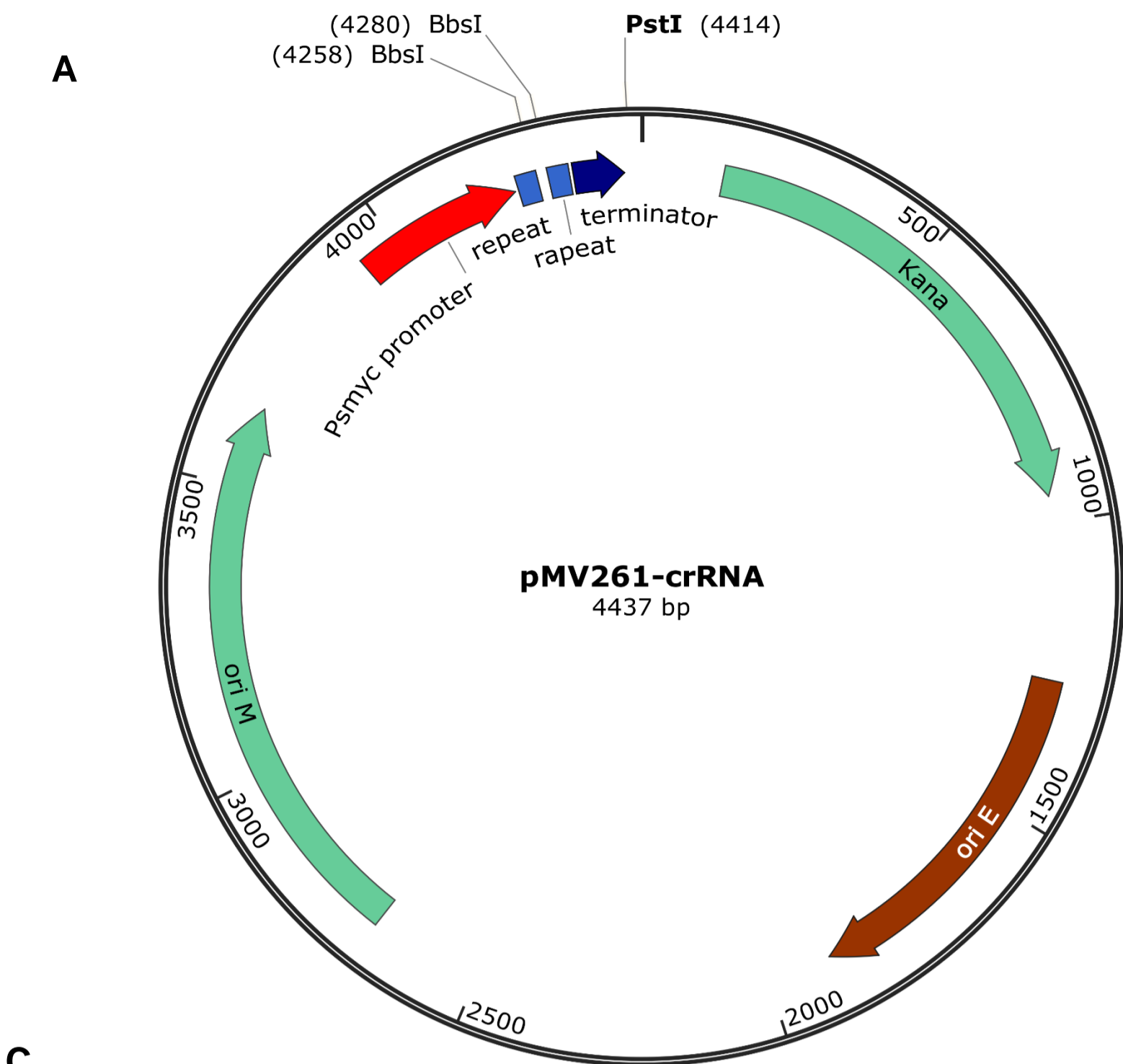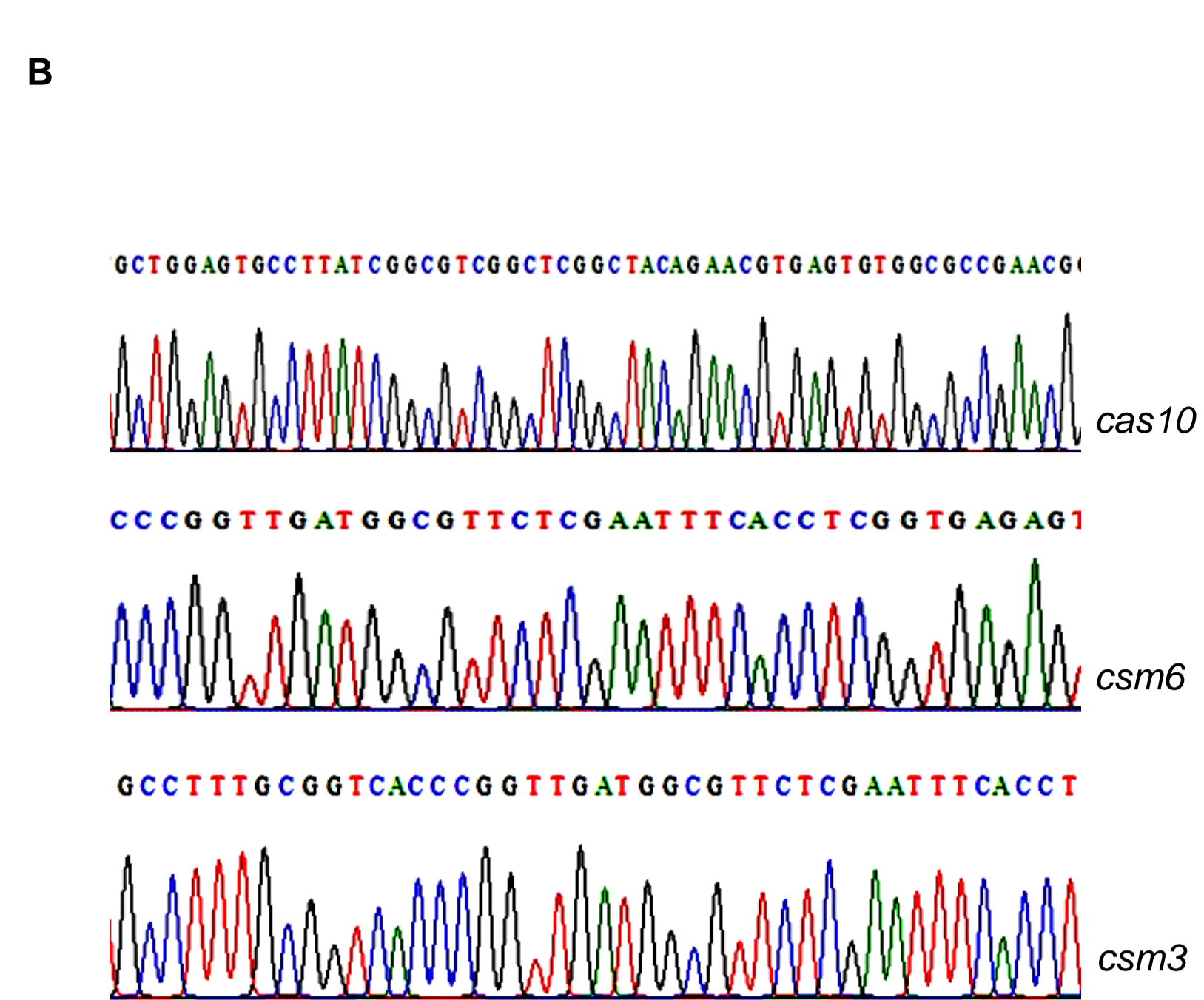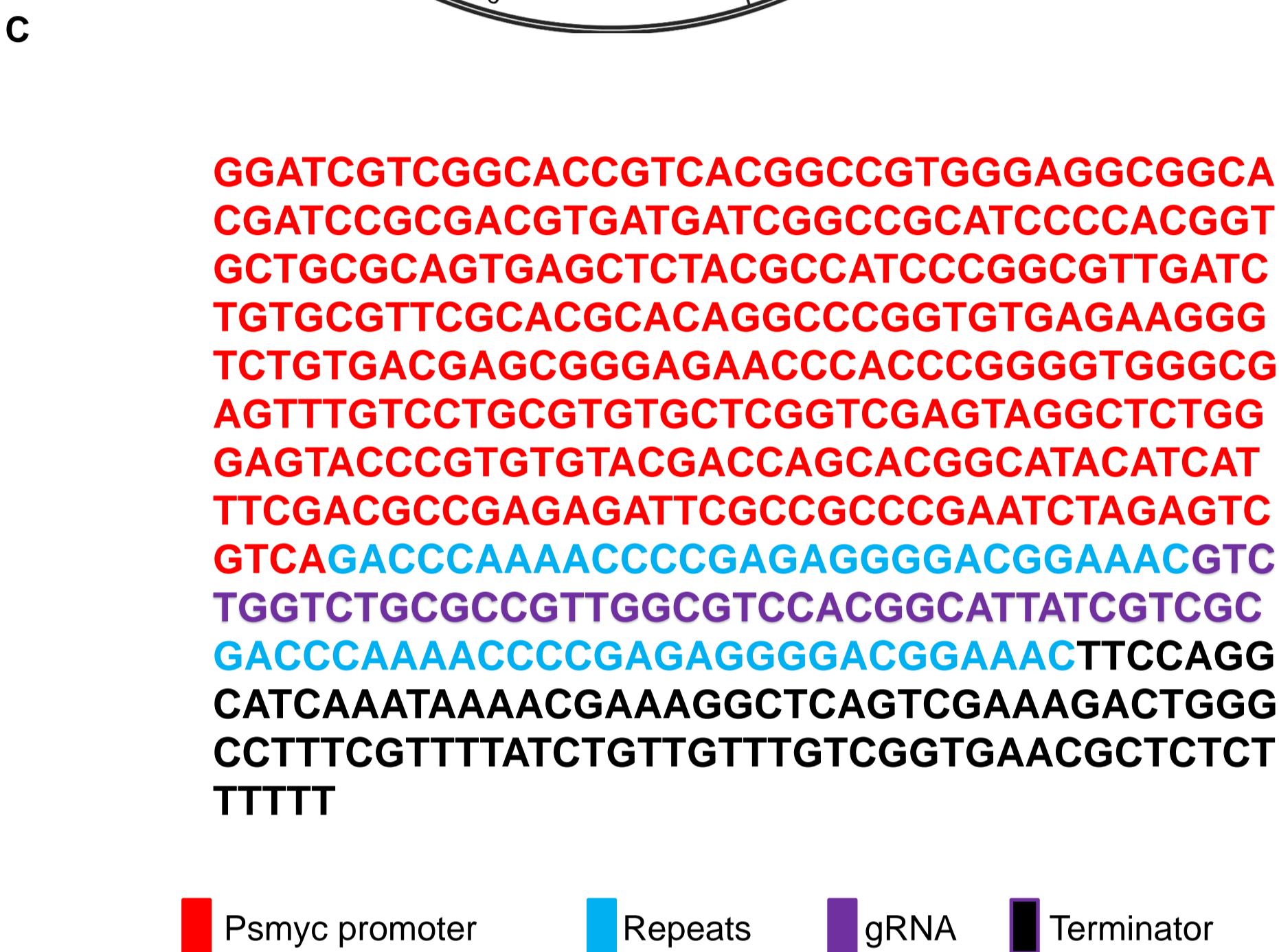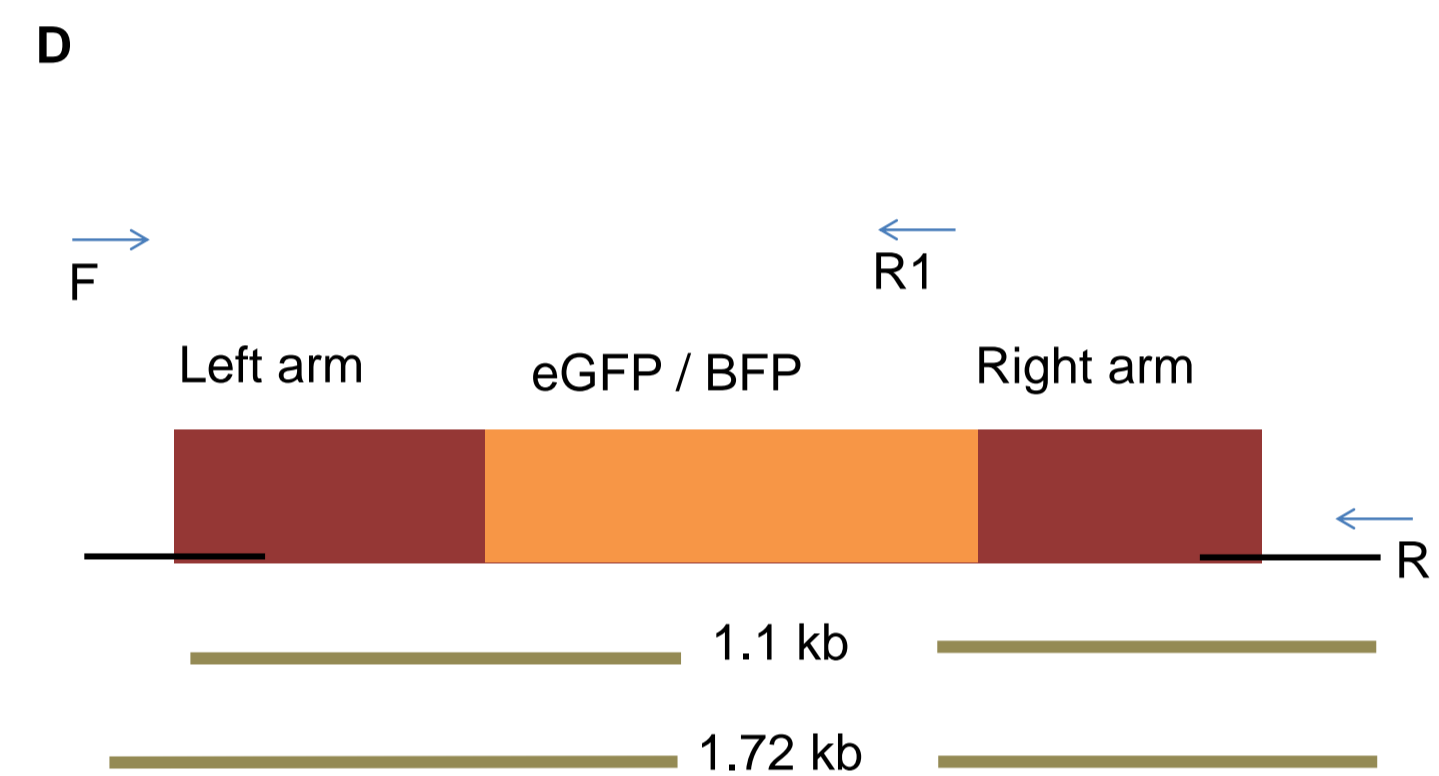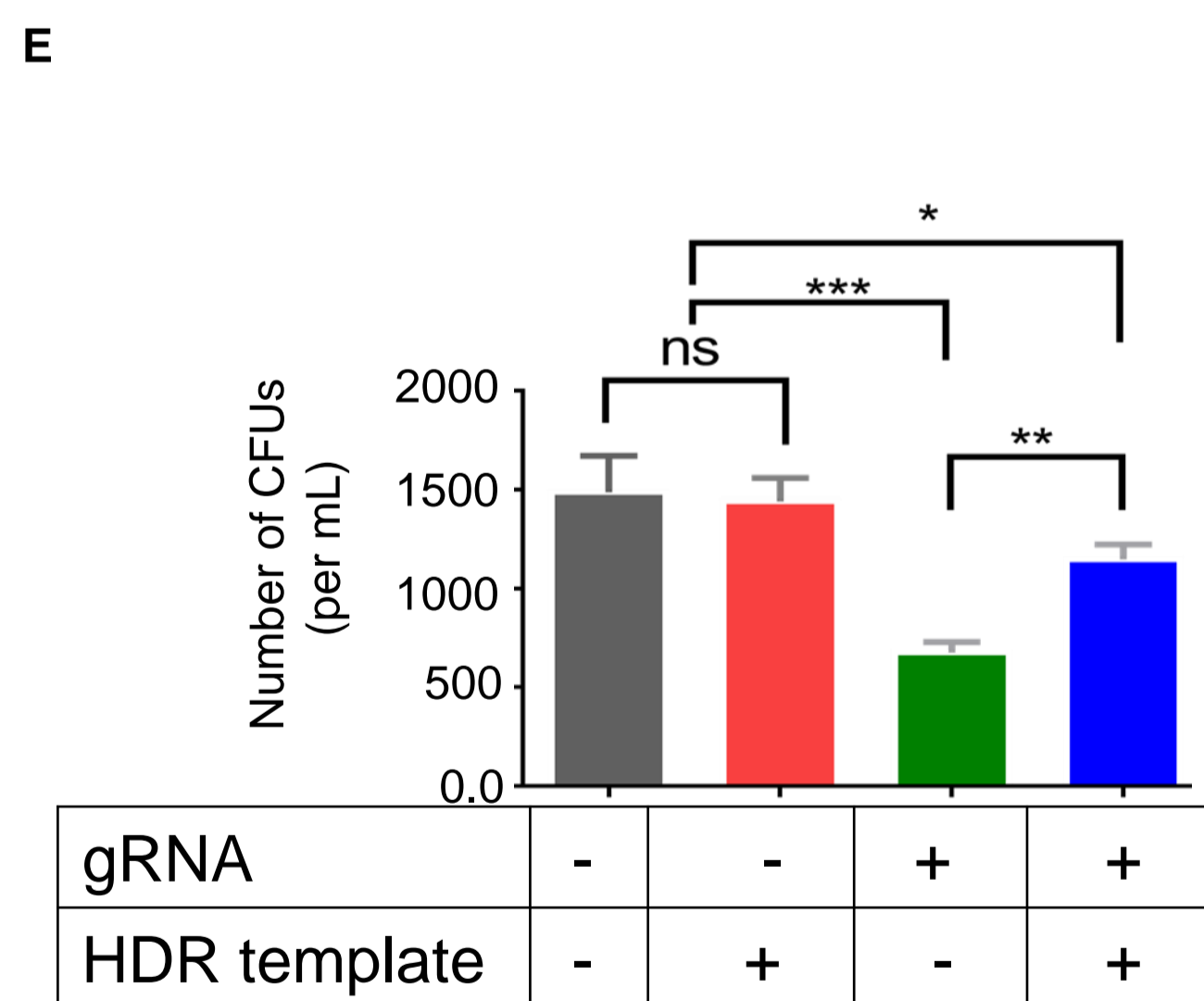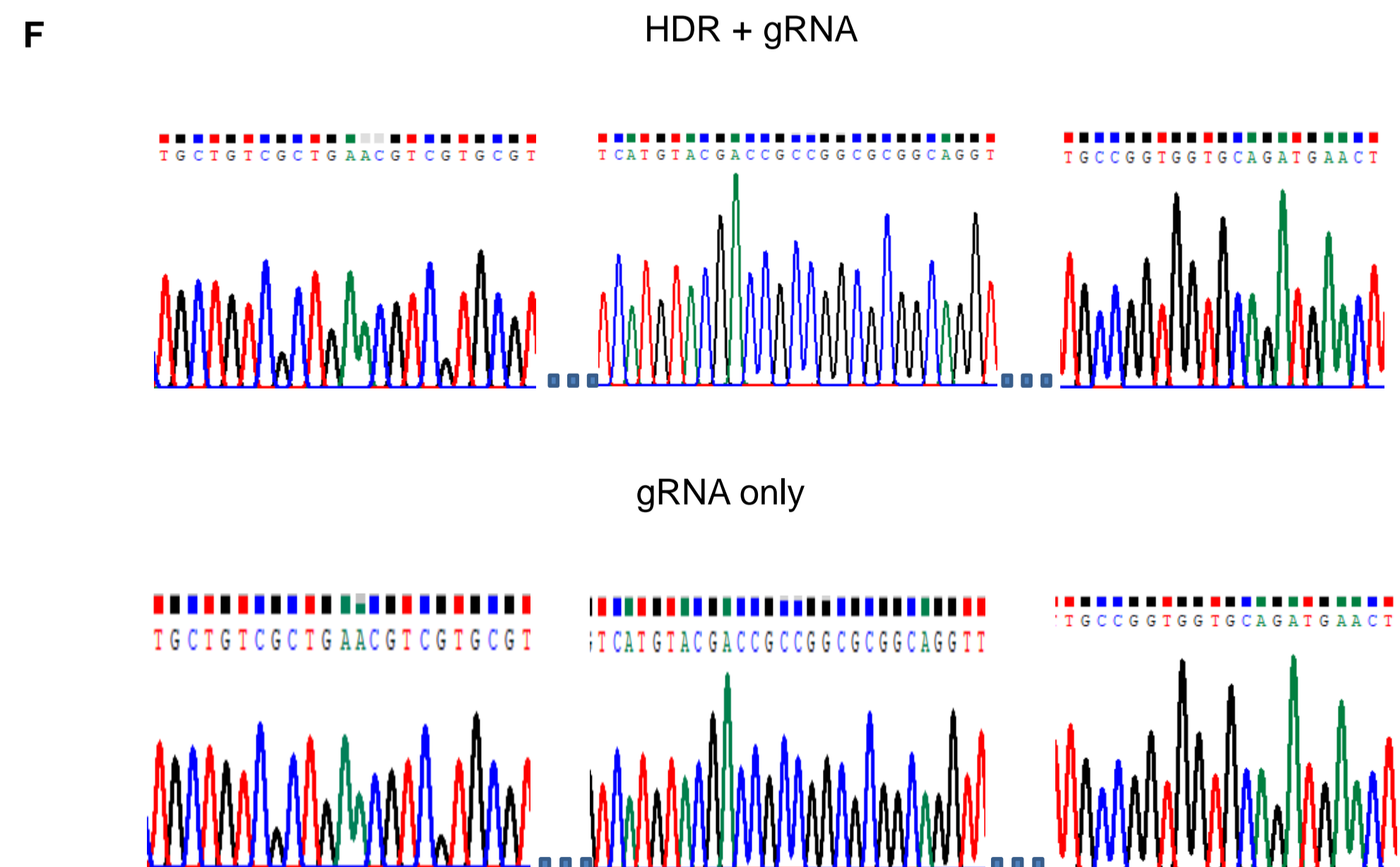

Supplement: Supplementary Figure S1 — Functional analysis of M. tuberculosis type III-A CRISPRS system A. Vector map of the construct for CRISPR-mediated gene editing and interference. B. Sanger sequencing chromatographs of qRT-PCR products of the respective cas/csm genes. C. Sequence information of Psmyc promoter, gRNA, repeats, and terminator. D. Cartoon representation of a general HDR template used for gene insertion and deletion. The black arrows with R and F labels on both ends of the HDR template represent the positions of the primers that could be used to confirm the insertion or deletion. E. Self-DNA is targeted by the endogenous type III-A CRISPR system directed by a gRNA designed for the gyrA gene. The graph represents the number of CFUs of M. tuberculosis transformed with gRNA, HDR, gRNA plus HHDR or empty plasmid. F. Sanger sequencing of the CFUs transformed with the HDR template and gRNA and those transformed with gRNA only. [file mmc1.pdf]

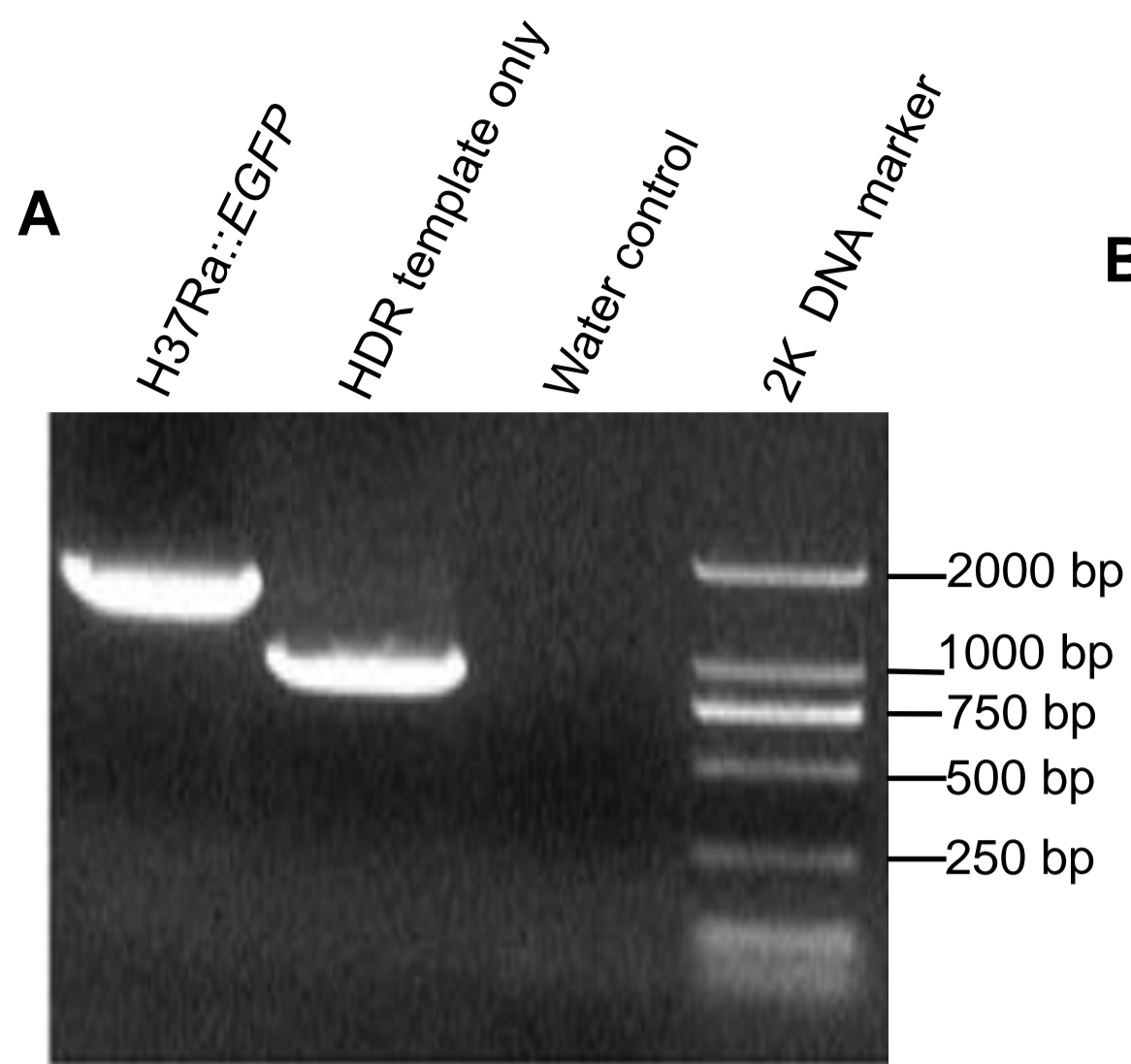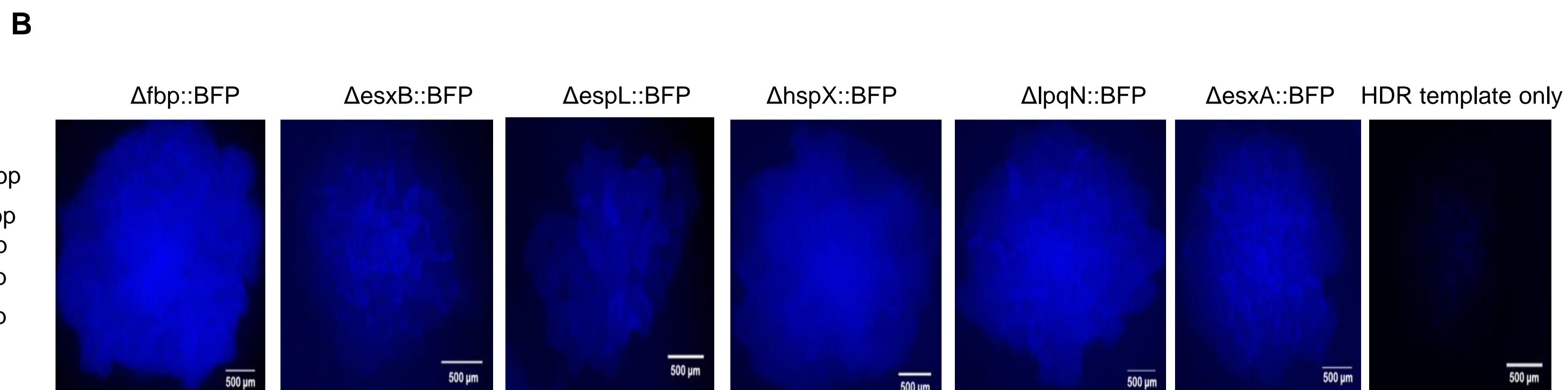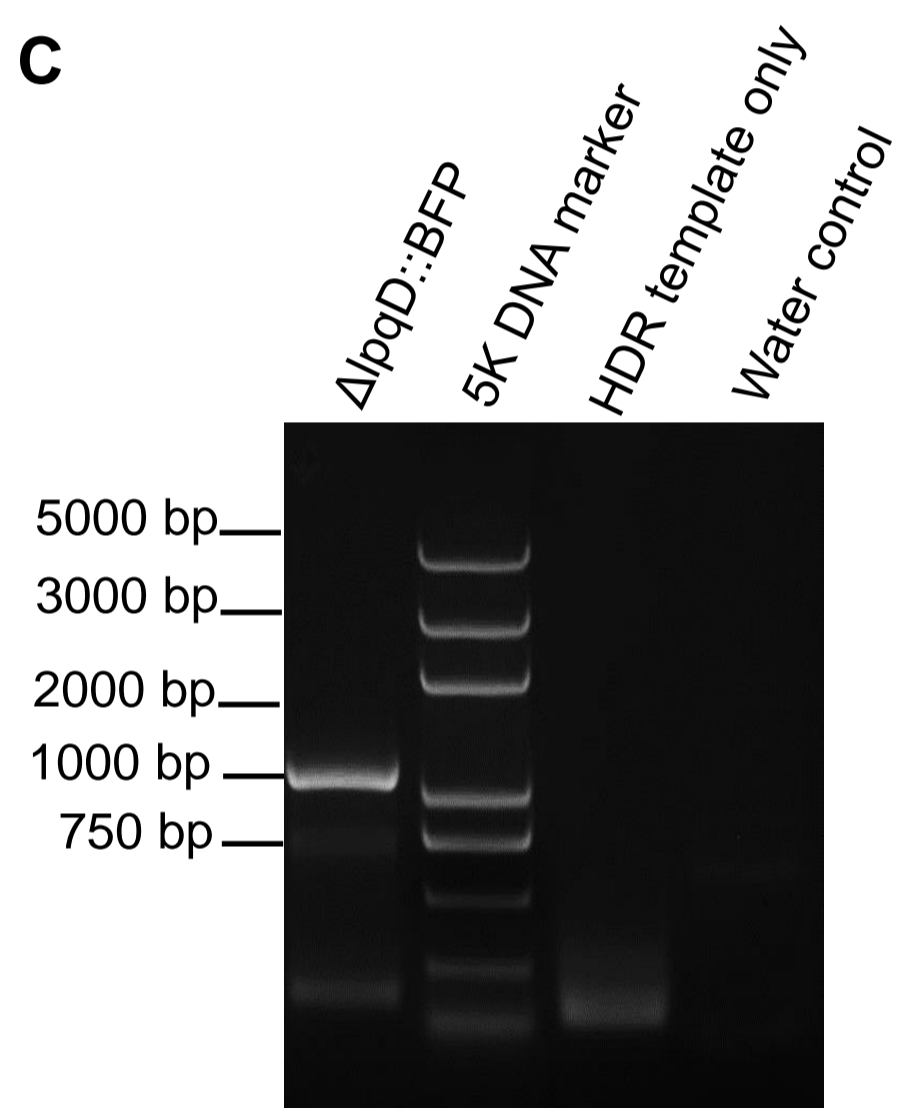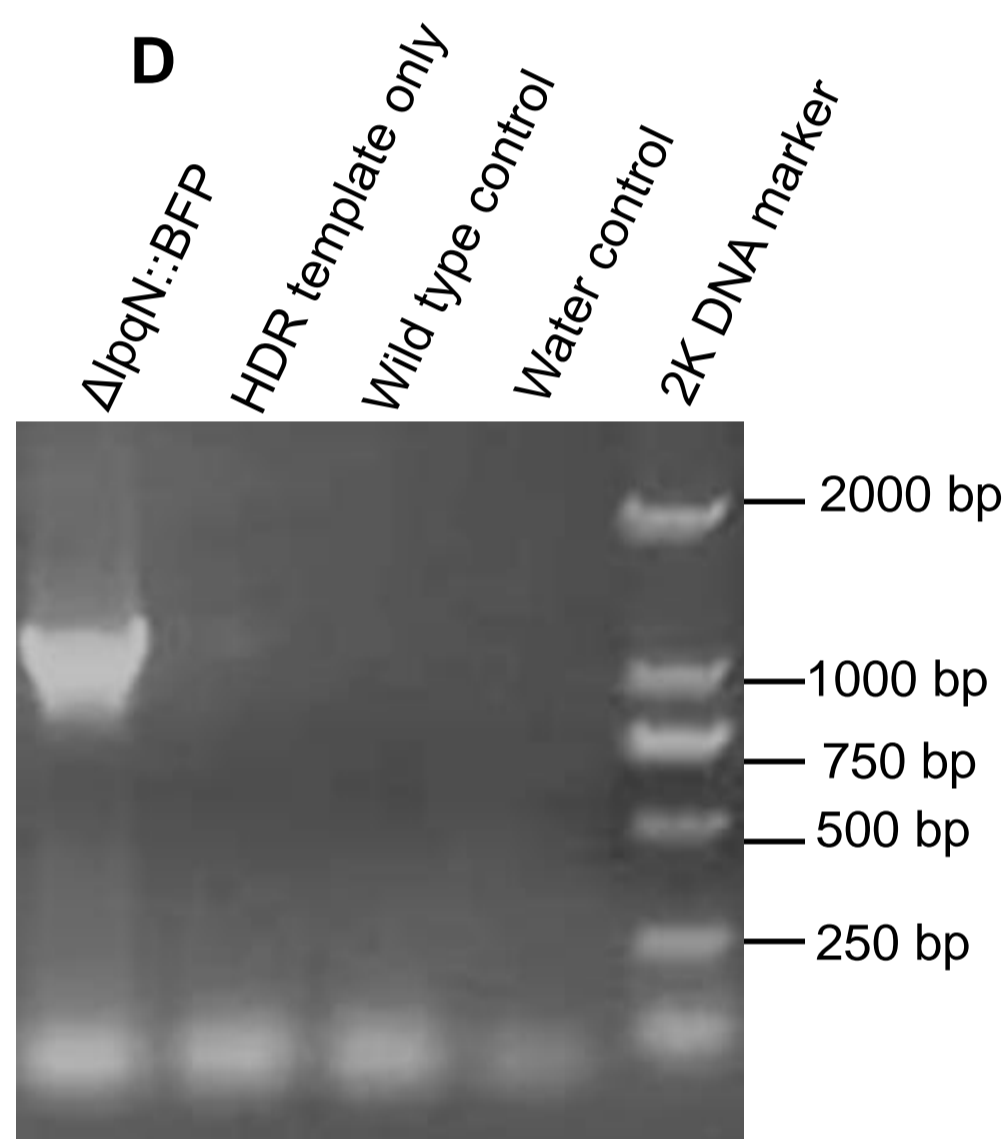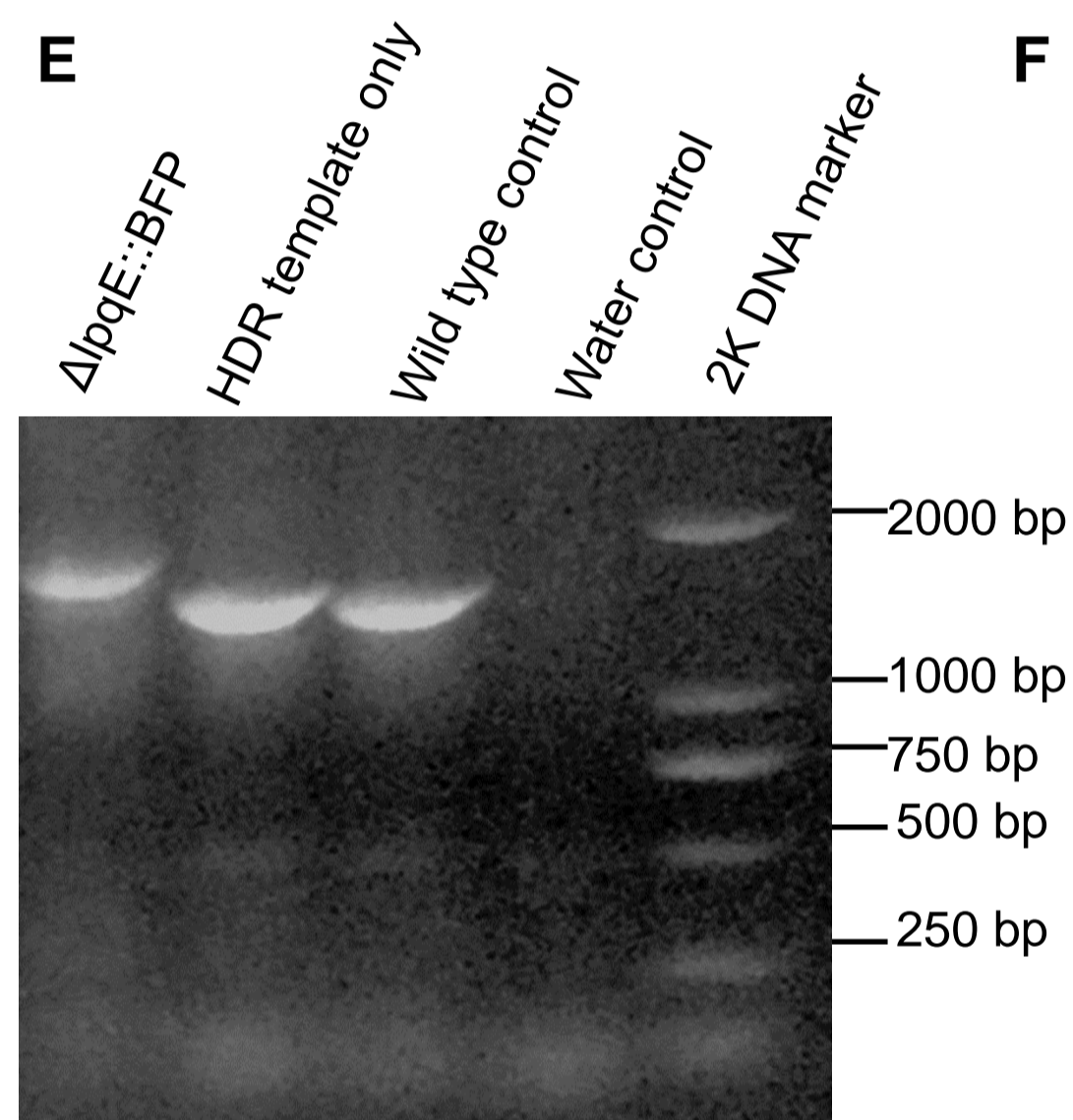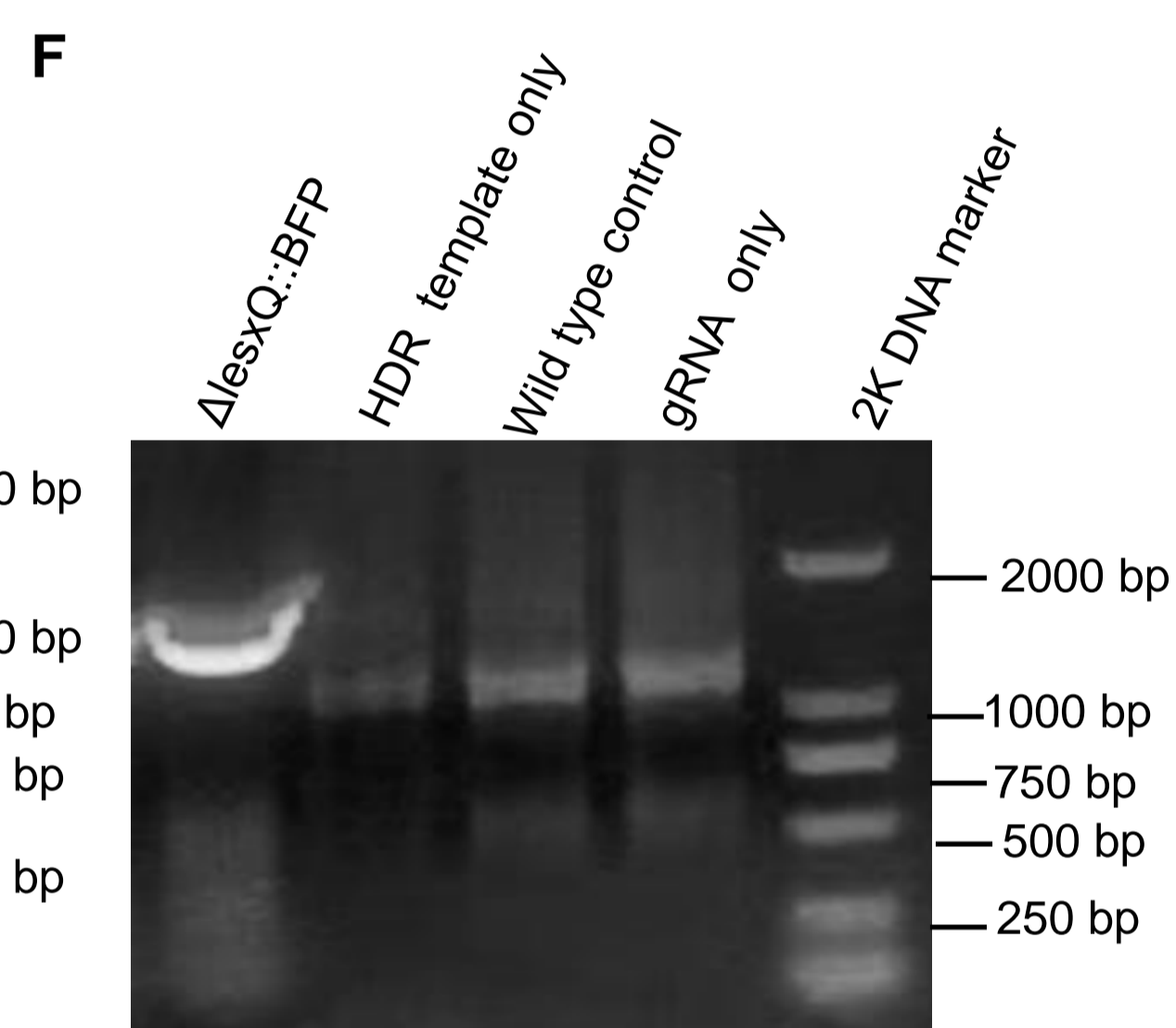

Supplement: Supplementary Figure S2 — Type III-A CRISPR system-mediated gene editing in M. tuberculosis. A. PCR confirmation of EGFP knock-in into the gyrA locus. B. Blue fluorescence in bacterial colonies expressing BFP after replacement of the target gene. C.–F. PCR confirmation of gene knockout. [file mmc2.pdf]

**A**

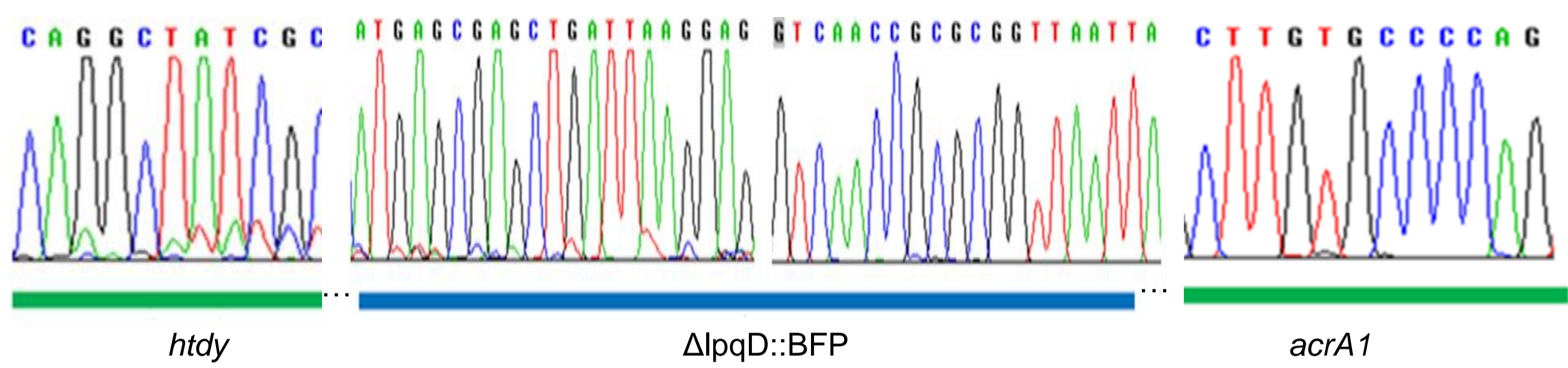

**B**

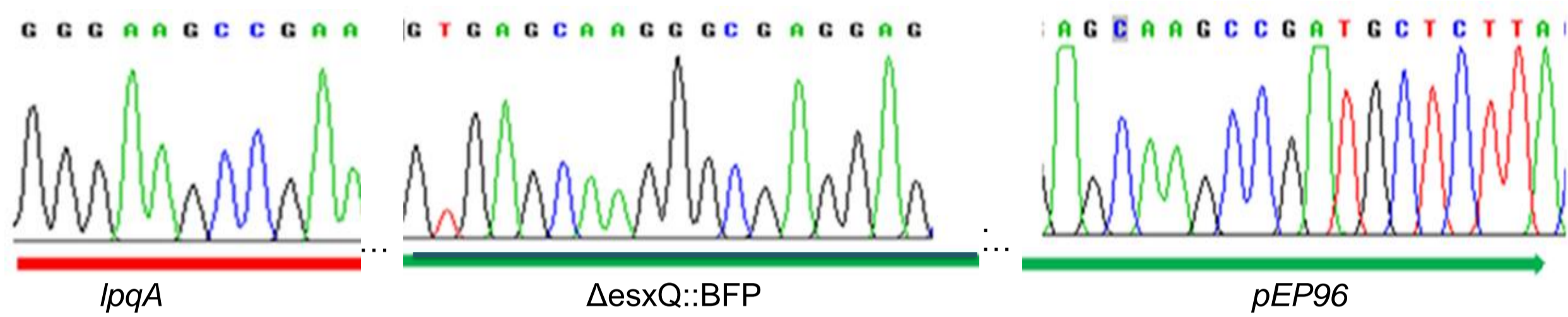

**C**

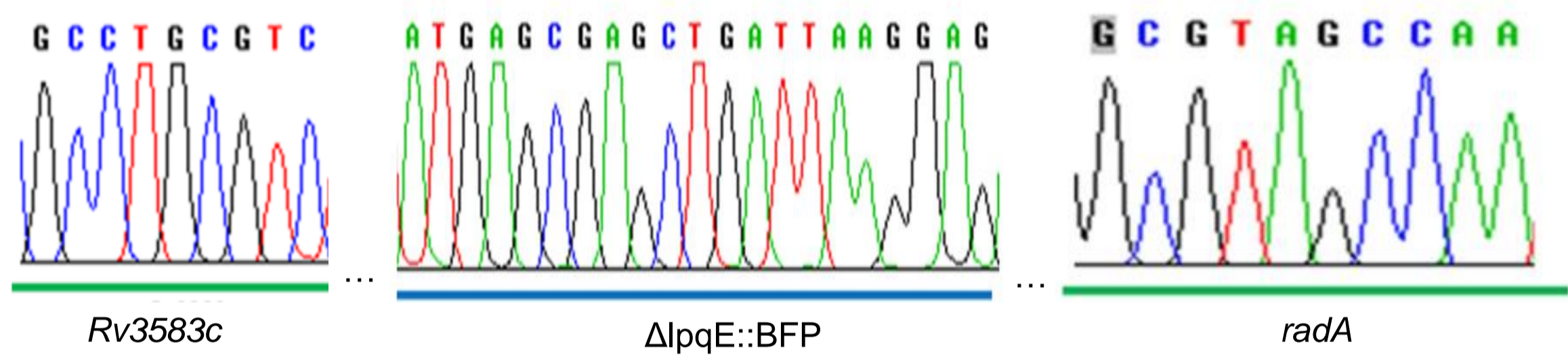

Supplement: Supplementary Figure S3 — Representative Sanger sequencing chromatographs revealing BFP insertion into the target sites. [file mmc3.pdf]

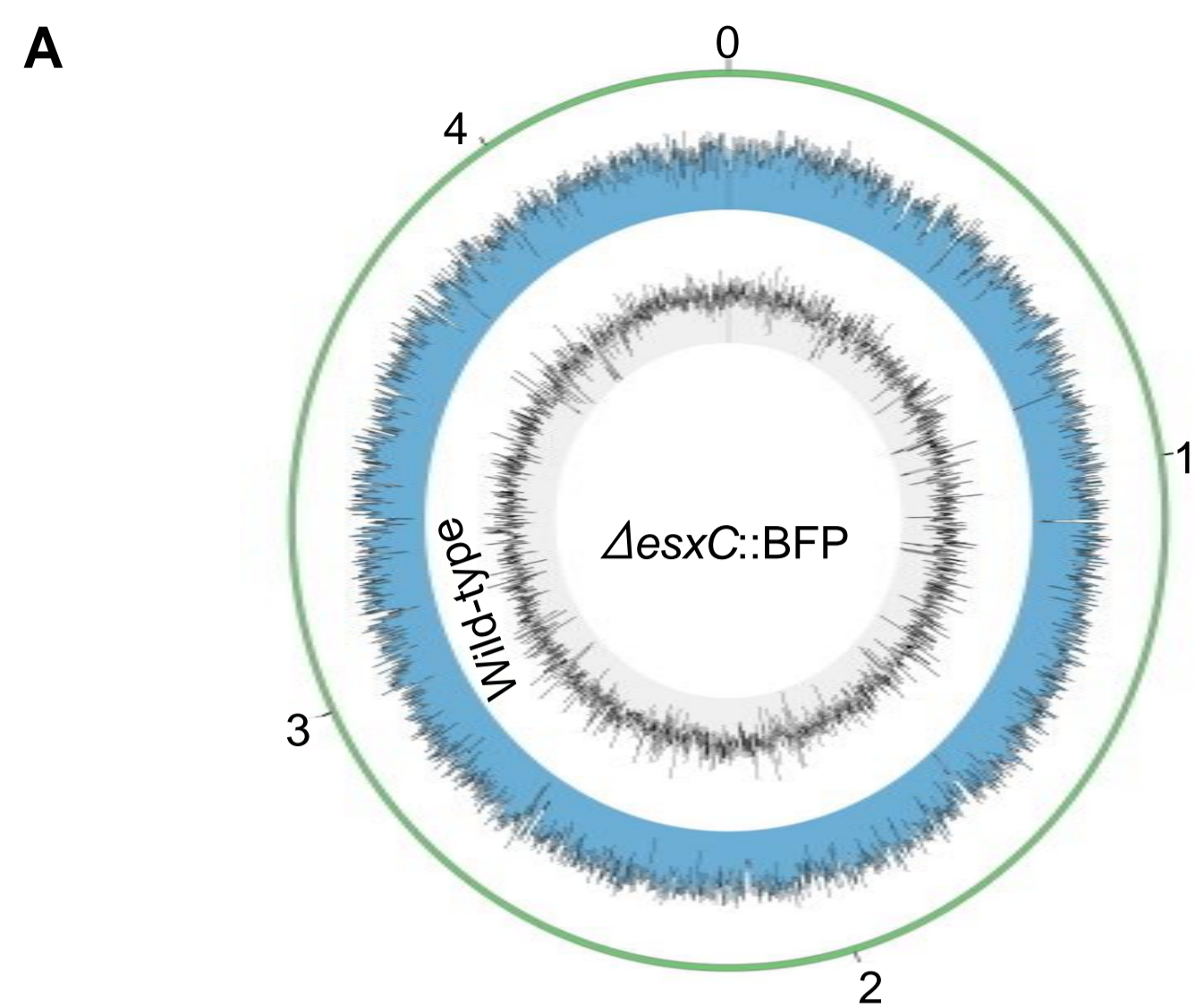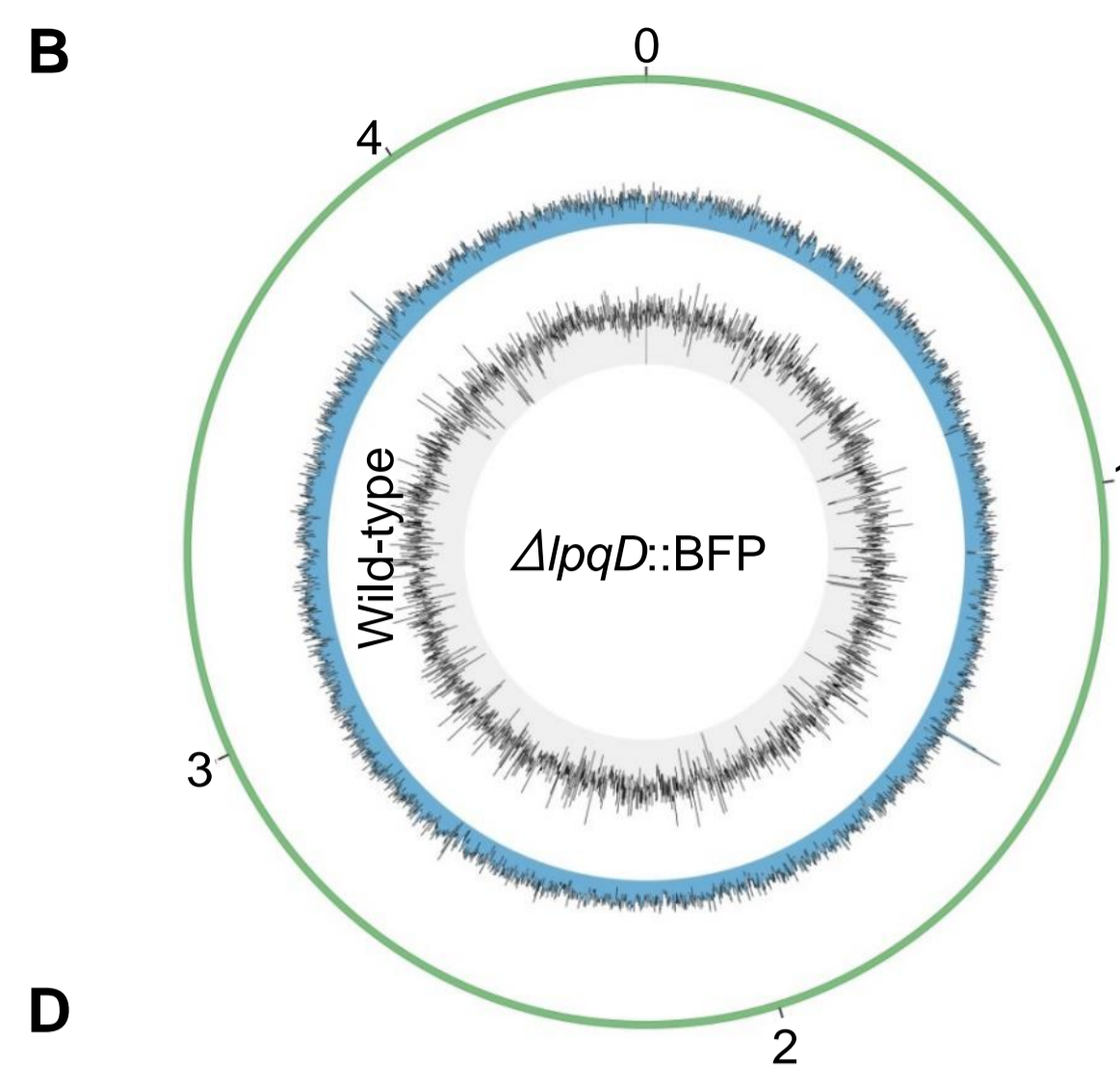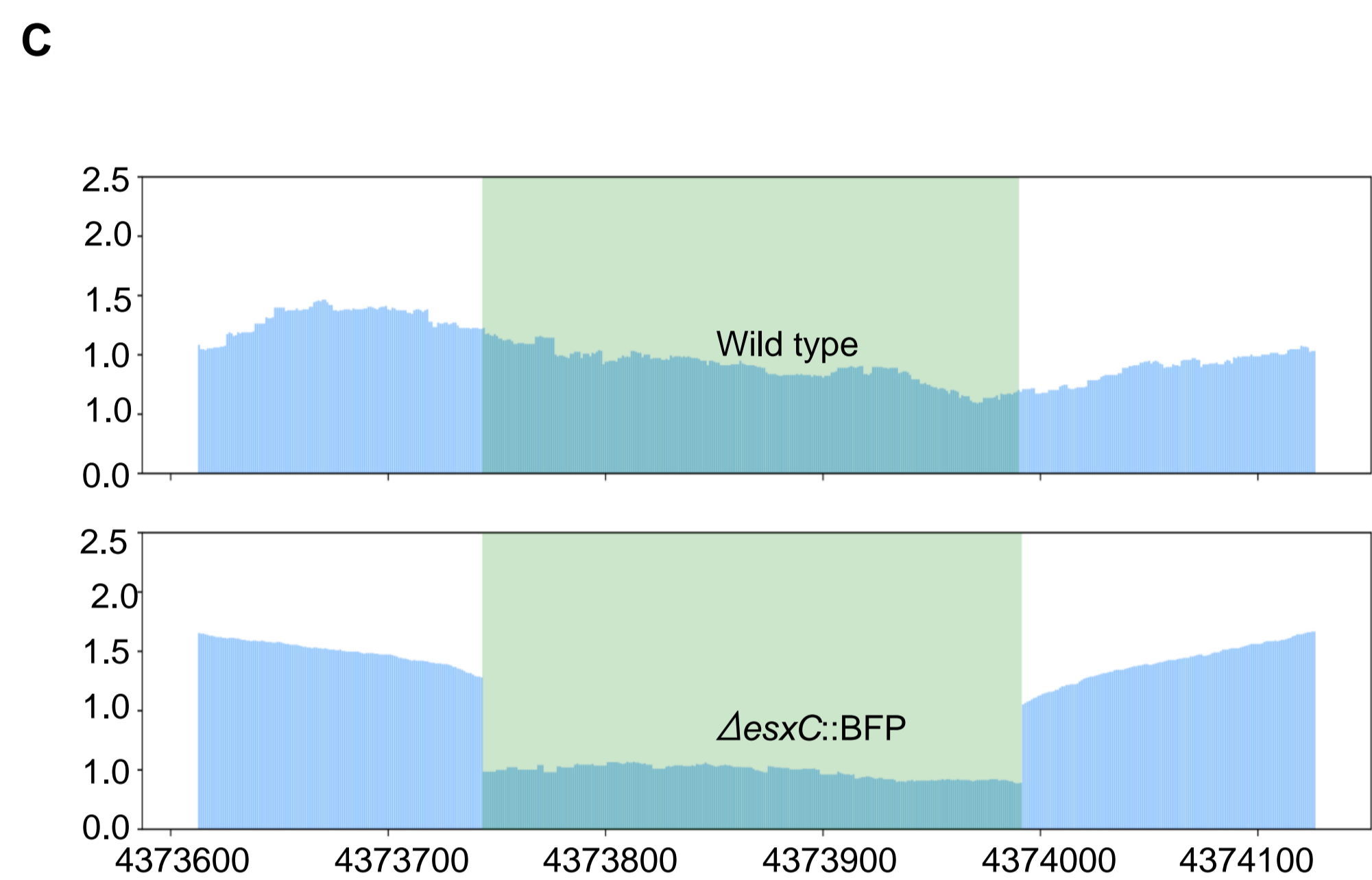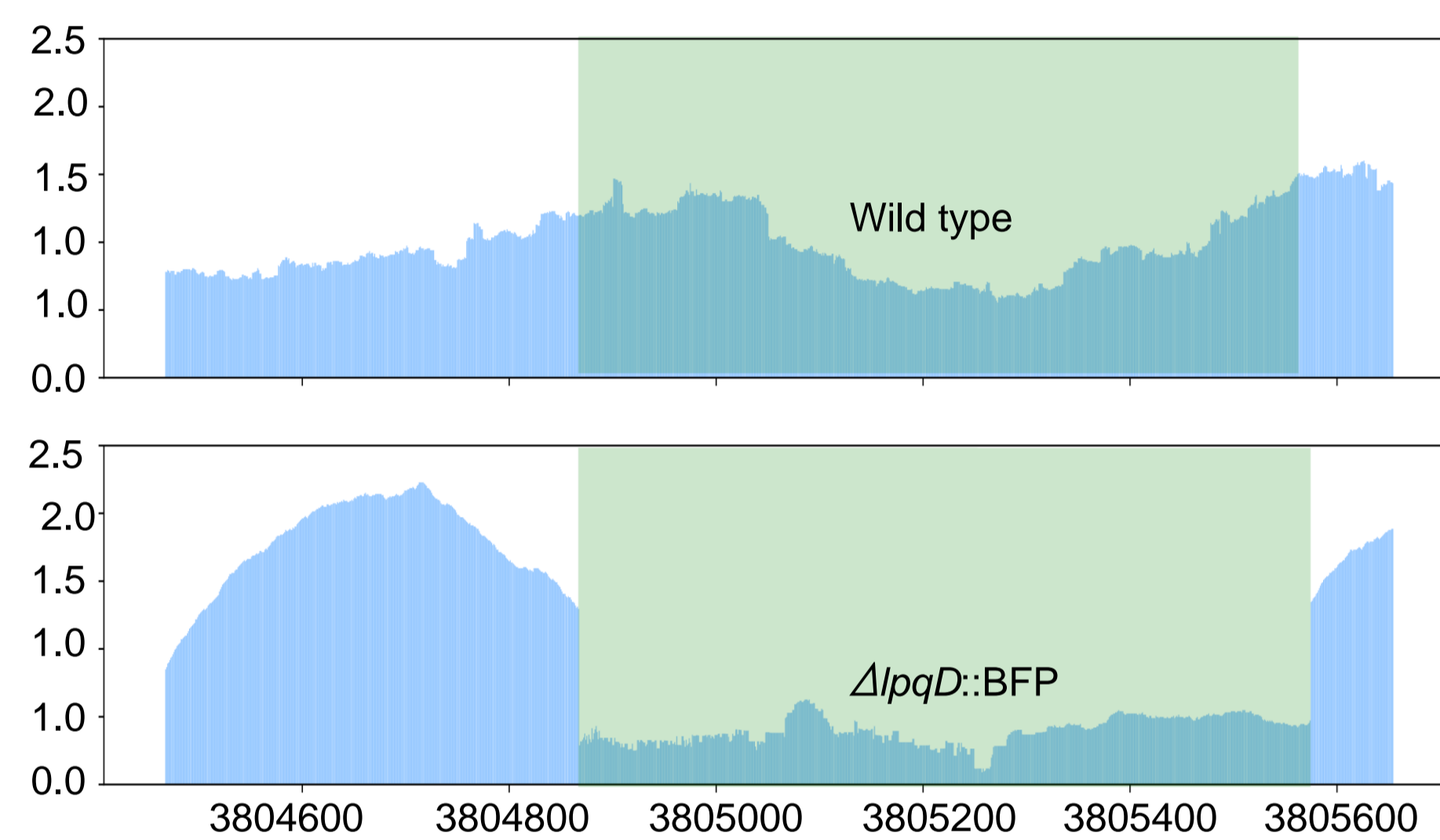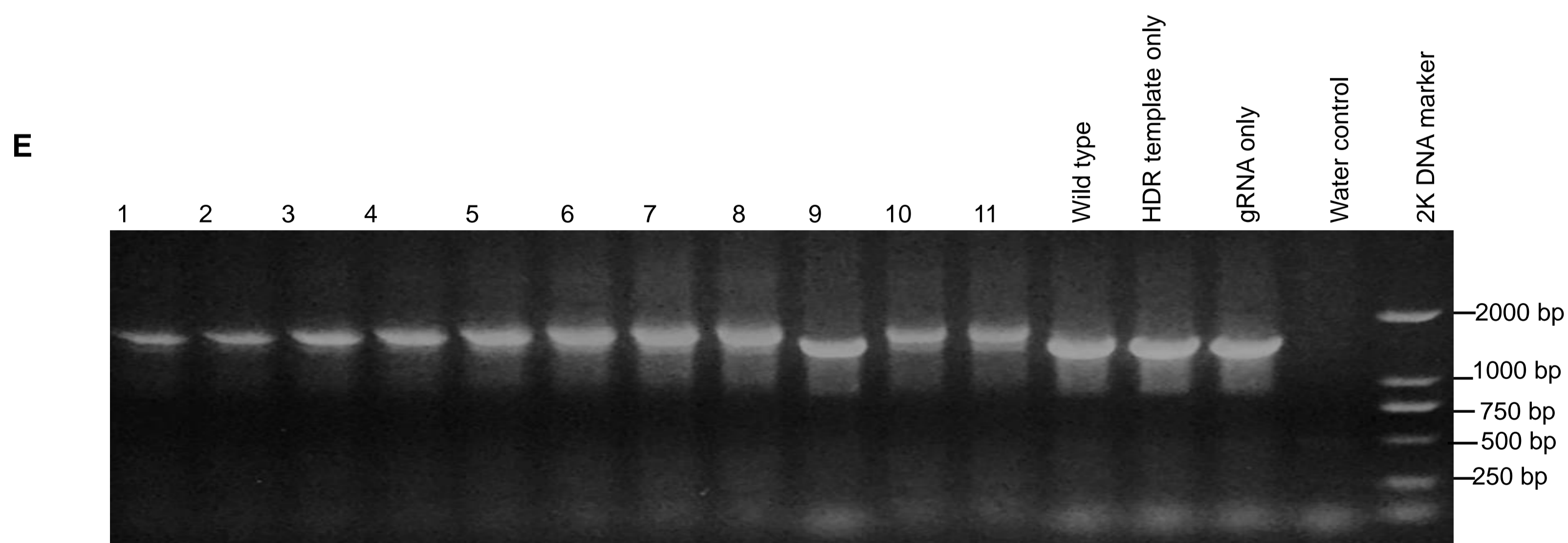

Supplement: Supplementary Figure S4 — Efficiency and off-target evaluation of type III-A CRISPR-mediated gene deletion A. and B. Circos plots showing the genomic outlines of the ΔesxC::BFP and ΔlpqD::BFP strains along with their wild-type strains. C. and D. Representation of the sequencing reads aligned on the esxC and lpqD locus in the wild-type and respective mutant strains. E. PCR amplification showing successful insertion of BFP in 10 out of 11 colonies. The bands on the gel from 1 to 11 represent H37Ra transformed with the plasmid containing the gRNA plus HDR template corresponding to lpqE gene. [file mmc4.pdf]

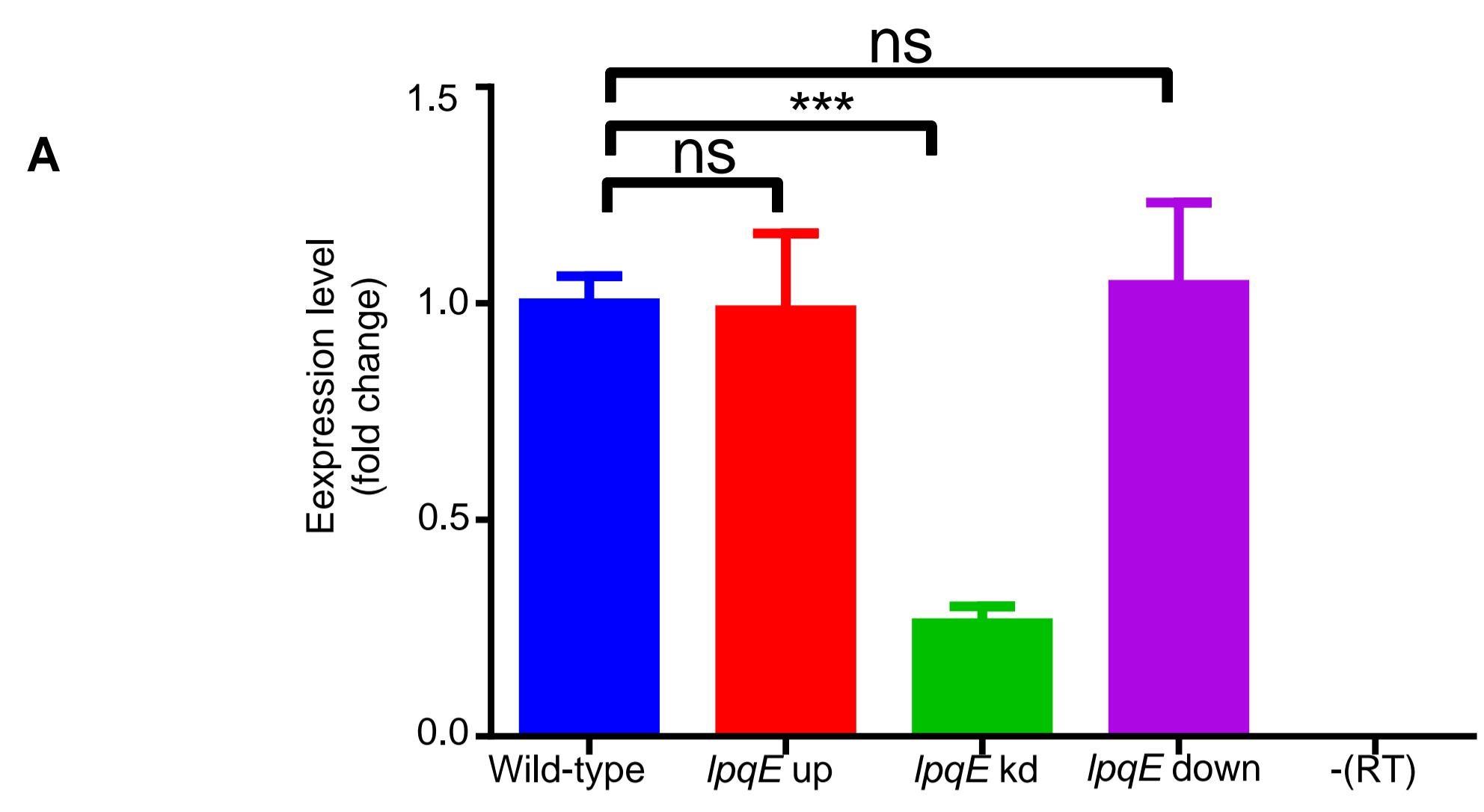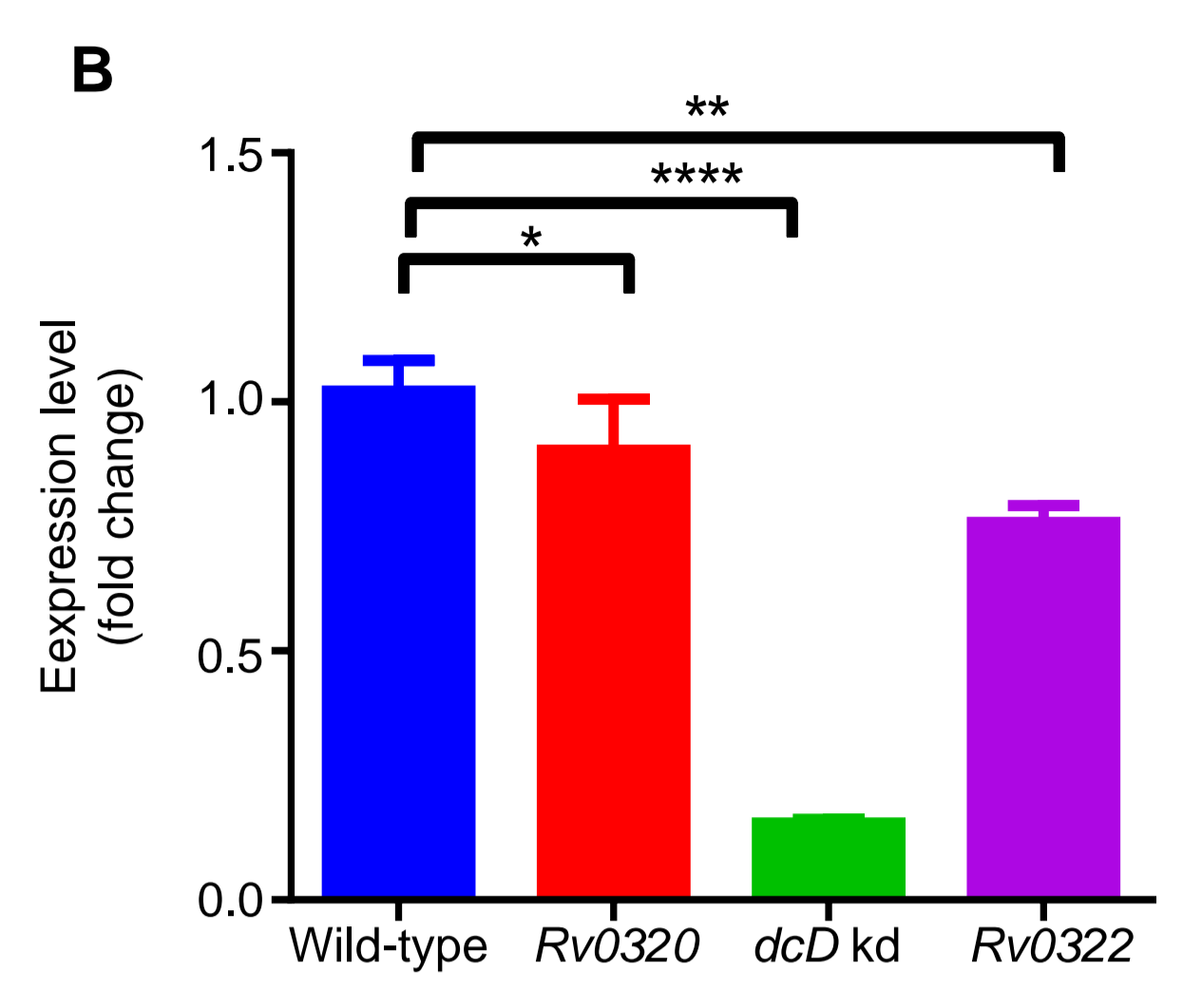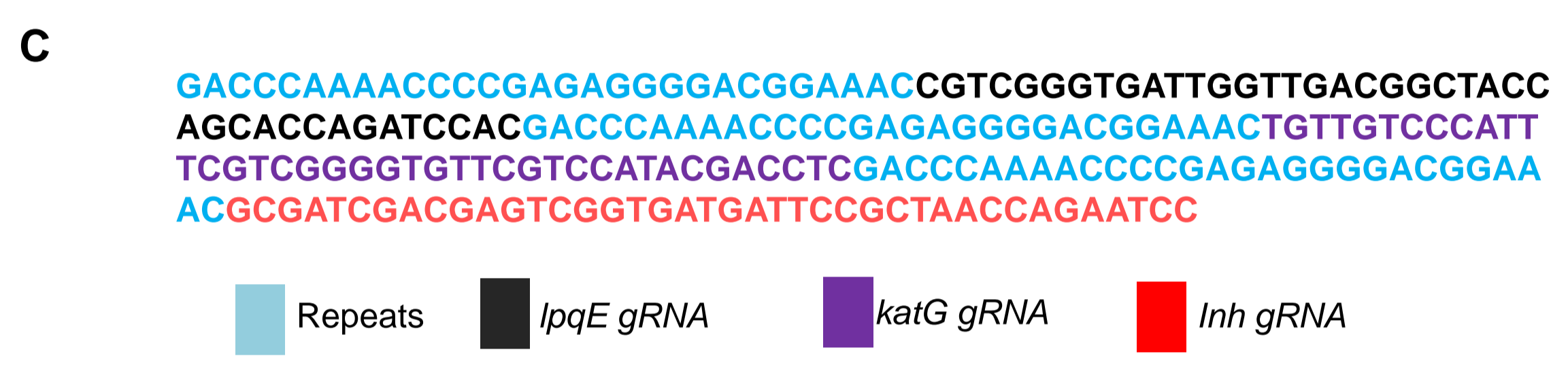

Supplement: Supplementary Figure S5 — Type III-A CRISPR-mediated RNAi in M. tuberculosis A. and B. Polar effects of the endogenous type III-A CRISPR-mediated RNAi on the neighbor genes of lpqE and dcD. C. Sequences of the spacers used for multiple-gene interference. [file mmc5.pdf]
