# Supplementary material for: Reprogramming Mycobacterium tuberculosis CRISPR System for Gene Editing and Genome-wide RNA Interference Screening
Source: Genomics Proteomics Bioinformatics. 2021 Dec 16;20(6):1180–96. doi: 10.1016/j.gpb.2021.01.008 (PMC10225669; doi:10.1016/j.gpb.2021.01.008)
Supplement: Supplementary Figure S6 — Strategy for gRNA library design for genome-wide RNAi screening in M. tuberculosis [file mmc6.pdf]

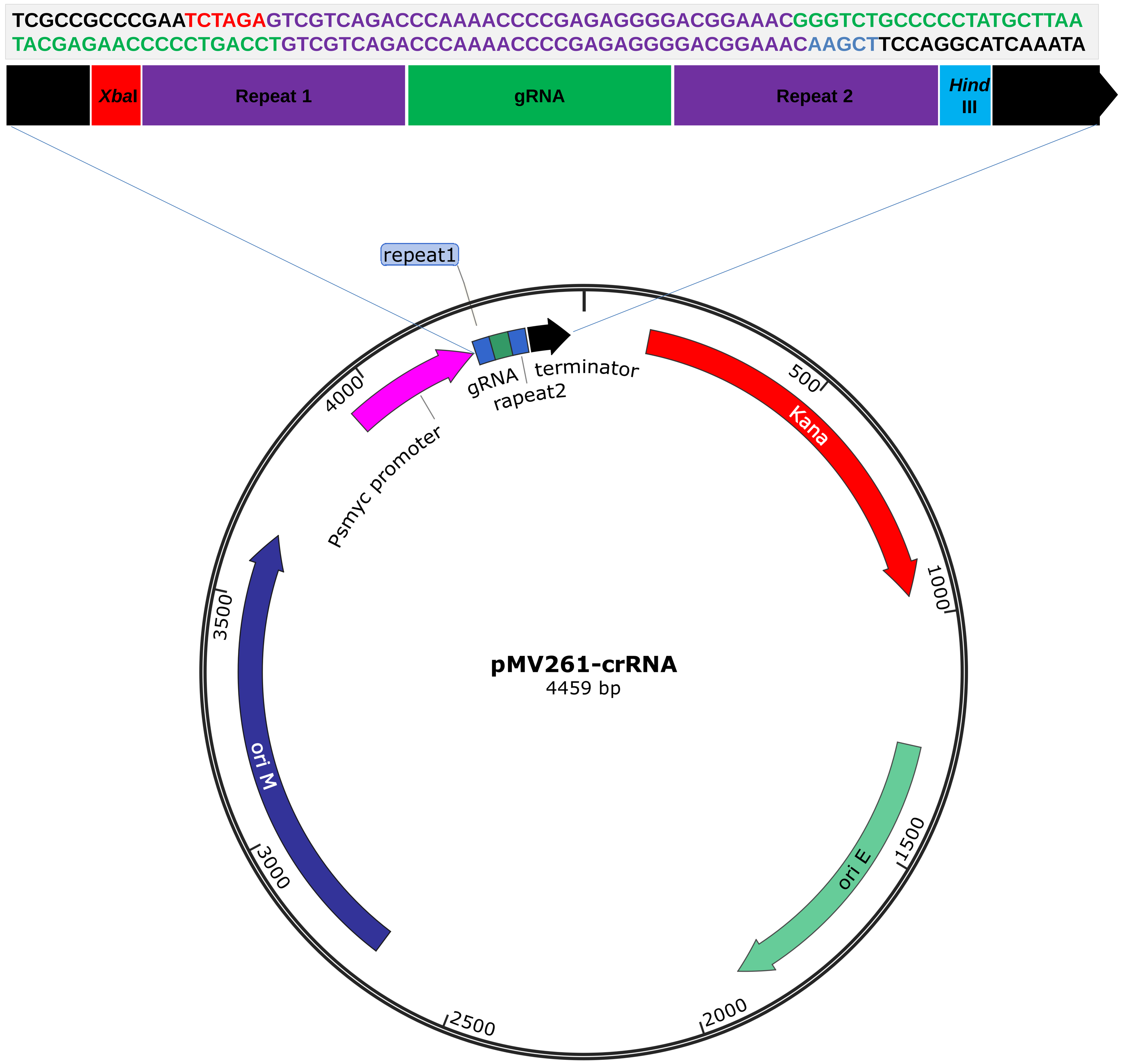

TCGCCGCCCGAATCTAGAGTCGTCAGACCCAAAACCCCGAGAGGGGACGGAAACGGGTCTGCCCCCTATGCTTAA
TACGAGAACCCCCTGACCTGTCGTCAGACCCAAAACCCCGAGAGGGGACGGAAACAAGCTTCCAGGCATCAAATA
XbaI
Repeat 1
gRNA
Repeat 2
Hind III
repeat1
gRNA
rapeat2
terminator
Psmyc promoter
Kana
ori E
ori M
500
1000
1500
2000
2500
3000
3500
4000
pMV261-crRNA
4459 bp
