# Supplementary material for: Reprogramming Mycobacterium tuberculosis CRISPR System for Gene Editing and Genome-wide RNA Interference Screening
Source: Genomics Proteomics Bioinformatics. 2021 Dec 16;20(6):1180–96. doi: 10.1016/j.gpb.2021.01.008 (PMC10225669; doi:10.1016/j.gpb.2021.01.008)
Supplement: Supplementary Table S1 — List of gRNA sequences used for gene knockout/knock-in and RNA interference [file mmc7.docx]

Table **S1 List of gRNA sequences used for gene knockout/knock-in and RNA interference**

| Primer name | Sequence 5'-3' | Position (bp) |
| --- | --- | --- |
| gRNA-*EGFP*-KI-*gyrA* | GTCTGGTCTGCGCCGTTGGCGTCCACGGCATTATCGTCGC | 2468-2507 |
| gRNA-*lpqE*-KO | CACCAGCCGGTCCGAAACGTCGGGTGATTGGTTGACGGCT | 246-285 |
| gRNA-*lpqN*-KO | GACGACGTCGACAACGACTACGTCGGCAACCACCTCGGCT | 117-156 |
| gRNA-*hspX*-KO | CGCGGACCATAATGTCGACGTCCTTGTCGGGGTCGACCCC | 222-261 |
| gRNA-*fbpB*-KO | CCGACGAGCCGGCCATCGACAAGCCGATTGCAGCGCTGCC | 465-504 |
| gRNA-*esxB*-KO | CTTCTGCTTATTGGCTGCTTCTTGGAAGCGCACCACCGCG | 159-198 |
| gRNA-*espL*-KO | GGCCGGTGAGCCACTGGTGCCCATTGATCGTCACTTCGAC | 177-216 |
| gRNA-*esxA*-KO | ACACCCTGGTACGCCTCCGAACCGCTACCGCCCCAGGCCG | 122-161 |
| gRNA-*esxQ* -KO | TGAGTCATCAGGACTGGCAGGCCCAGTGGAATCAGGCCAT | 145-184 |
| gRNA-*lpqD*-KO | TGATTCGGGTTTGCCGTTGAACCAGCCGGCGTTGATCGCT | 345-384 |
| gRNA-*esxC*-KO | GACGTGGGCTCGCGCGCCGGCCAGCTCCACATGATTTACG | 51-90 |
| gRNA-*katG*-KD | GAAACTACCTCGGAAAGGGCAACCCGTTGCCGGCCGAGTACATGC | 1228-1272 |
| gRNA-*esxT*-KD | GAAACGGGCCGCGGTGGTGTGGATCTCCTGACGAACGGAGTATTC | 217-261 |
| gRNA-*dcd*-KD | GAAACAAGCAATCGAGCCGGACGTCGATGCTGGACGGCTGGACCA | 72-116 |
| gRNA-*InhA*-KD | GAAACGCGATCGACGAGTCGGTGATGATTCCGCTAACCAGAATCC | 21-65 |
| gRNA-*lpqN*-S2-KD | GAAACTGCAGCCAGCCAGTGCCAGGCTCAGCGCCACCGTCGCGAC | 621-665 |
| gRNA-*lpqN*-S1-KD | TTCAACTCGCCGGGTGCGAATTGGAGGACCTTCGCGGGGT | 250-289 |
| gRNA-*lpqE*-KD | GAAACCGTCGGGTGATTGGTTGACGGCTACCAGCACCAGATCCAC | 228-272 |
| gRNA-*dcD*-KD | GAAACTGGCCCGGCATGAAAATCGGTCAGCTGTGCATGTTGCGCC | 421-465 |
