# Supplementary material for: Reprogramming Mycobacterium tuberculosis CRISPR System for Gene Editing and Genome-wide RNA Interference Screening
Source: Genomics Proteomics Bioinformatics. 2021 Dec 16;20(6):1180–96. doi: 10.1016/j.gpb.2021.01.008 (PMC10225669; doi:10.1016/j.gpb.2021.01.008)
Supplement: Supplementary Table S2 — List of primers for gene knockout, knock-in, knockout, and qRT-PCR analysis [file mmc8.docx]

**Table S2 List of primers for gene knock-in, knockout, and qPCR analysis**

| Primer name | Sequence 5′-3′ |
| --- | --- |
| *gyrA*-KI-left arm-F | CAATATCGACGACCGGCTGCTGTCGCT |
| *gyrA*-KI-left arm-R | ATTGCCCGTCTGGTCTGCGCCGTTGGC |
| *gyrA*-KI-right arm-F | TCAGGCTCGCCCGACGACGATGCGGAT |
| *gyrA*-KI-right arm-R | CGGGGTCCTGACCGCGGCGGCTGGGCC |
| *gyrA*-KI-EGFP-F | CAGACGGGCAATGTGAGCAAGGGCGAGGAGC |
| *gyrA*-KI-EGFP-R | GGGCGAGCCTGATTACTTGTACAGCTCGTCCA |
| *gyrA*-KI-detection-F | CTCCGCGGGACGCGCCGGCGCGGGGTTGCG |
| *gyrA-*EGFP-detection-R | CCCCTTTGATGGTCCGGGTTTCGATCGCCT |
| *lpqE*-KO-left arm-F | TTGGCTACGCCGCACGAATAGGCGCCGCA |
| *lpqE*-KO-left arm-R | CAACAGCGTCGACTTACCCACTCCGGGAT |
| *lpqE*-KO-right arm-F | CCTGCGTCTTATGAGCGAGCTGATTAAGG |
| *lpqE*-KO-right arm-R | CGGCGTAGCCAATTAATTAAGCTTGTGCCCCA |
| *lpqE*-BFP-F | GACGTATTCGGCGTTTTCAGCGGGAACTCG |
| *lpqE*-BFP-R | CGGATCTGACCGGCGGATTCCTCACCAGAG |
| *lpqE*-KO-detection-F | CTTGTCCTGGATGTCGGCGACCTCGGTGAC |
| *lpqE*-KO-detection-R | GGCGATAGCCTGCCCTGTCGATACCGTC |
| esx*Q*-KO-left arm-F | CGACGATTTCGCCCAACCCATACCACG |
| esx*Q*-KO-left arm-R | AAACTGGGGCACAAGCTTAATTAACTAACCCGGCACGTTG |
| *esxQ*-KO-right arm-F | CTCCTTAATCAGCTCGCTCATGTGACGGCGC |
| *esx*Q-KO right arm-R | CACGATCCCGATCGCGCTCAACGAGGCCGACTAT |
| *esxQ*-BFP-R | CAACGTGCCGGGTTAGTTAATTAAGCTTGTGCCCCAGTTTGC |
| *esxQ*-BFP-F | GCATGTTCTCCTTAATCAGCTCGCTCATGTGACGG |
| *esxQ*-KO-detection-F | CGGCAGTCTTTGGCTCTGAGATCGAAGCG |
| *lpqD*-KO-left arm-f | CCGCGCGGTTGACGCTTACCGCCGCTGAC |
| *lpqD*-KO-left arm-R | AGTCTCCGGCCACCGCGATCGACGACACG |
| *lpqD*-KO-right arm-F | AGGCTATCGCCATGAGCGAGCTGATTAAGGAG |
| *lpqD*-KO-right arm-R | TCAACCGCGCGGTTAATTAAGCTTGTGCCCC |
| *lpqD*-BFP-F | AGCGTTTCGGCAACCAACGAACCCGATTCC |
| *lpqD*-BFP-R | GTAAGCGTCAACCGCGCGGTCAGGTGAAGT |
| *lpqD*-KO-detection-F | CGCATCTACCGCCCGGCGGTGGTGGTGGGTGATTCG |
| *lpqN-*KO-detection-F | CACTTCACGGCGGCCGTCGCGAC |
| Gene KO detection primer R1 | TTAATTAAGCTTGTGCCCCAGTTTG |
| *katG*-qPCR-F | TTGCCCCAATAGACCTCATC |
| *katG*-qPCR-R | GCCGGTCAAGAAGAAGTACG |
| *esxT*-qPCR-F | GCCGGTCAAGAAGAAGTACG |
| *esxT*-qPCR-R | CCCAAGTCGATCAGGATCTC |
| *inhA*-qPCR_F | GGCAGAGTGAAAAGCTCCAG |
| *inhA*-qPCR-R | ATCCACATCTCGGCGTATTC |
| *lpqN*-qPCR-F | AGTTTCAACATCAAGACCG |
| *lpqN*-qPCR-R | TTGGCGGCTGCTTCCGGAAAG |
| *lpqE*-qPCR-F | ATTTCATCCAGCCAGGCAAAG |
| *lpqE*-qPCR-R | ATGCTTTTTGTCGGGACGCC |
| Pooled gRNA amp-F | CCCCGAGAGGGGACGGAAAC |
| Pooled gRNA amp-R | GGGGTTTTGGGTCTGACGAC |
| Library-Seq-F | CGACTAGTGACGCTCGTCGGGGAG |
| Library-Seq-R | ACGTGTACTTCGACCTGGATCGGG |
| Csm6-F | CTCAAGCACCCTCCGAAATC |
| Csm6-R | GCGAGGATCTTCAGCAGTTG |
| Csm3-F | CCATATCCGTCGGCTTTTCG |
| Csm3-R | ACAAGTGAGAACGCGAACTC |
| Cas10-F | GCCGCTTCCTACTCATCTCT |
| Cas10-R | AACTCCATCTGTGCGACGTA |
|  |  |
