# Supplementary material for: Reprogramming Mycobacterium tuberculosis CRISPR System for Gene Editing and Genome-wide RNA Interference Screening
Source: Genomics Proteomics Bioinformatics. 2021 Dec 16;20(6):1180–96. doi: 10.1016/j.gpb.2021.01.008 (PMC10225669; doi:10.1016/j.gpb.2021.01.008)
Supplement: Supplementary Table S5 — List of M. tuberculosis drug target genes based on in vitro screening method [file mmc11.docx]

T**able S5 List of *M. tuberculosis* drug target genes based on *in-vitro* screening method**

| Similarity score | Gene |
| --- | --- |
| 0.390227 | MRA_2157 |
| 0.401432 | MRA_3144 |
| 0.419705 | MRA_3158 |
| 0.426932 | MRA_1126 |
| 0.435364 | MRA_0579 |
| 0.439614 | MRA_3212 |
| 0.44125 | MRA_3216 |
| 0.442682 | MRA_1277 |
| 0.459455 | MRA_0648 |
| 0.462523 | MRA_1554 |
| 0.465318 | MRA_0678 |
| 0.465477 | MRA_3788 |
| 0.467977 | MRA_3175 |
| 0.47075 | MRA_3713 |
| 0.472068 | MRA_0363 |
| 0.472545 | MRA_1920 |
| 0.473659 | MRA_0057 |
| 0.478341 | MRA_1589 |
| 0.479727 | MRA_3401 |
| 0.481636 | MRA_3620 |
| 0.482977 | MRA_3791 |
| 0.483409 | MRA_1306 |
| 0.486682 | MRA_2816 |
| 0.489841 | MRA_0050 |
| 0.492455 | MRA_0325 |
| 0.493477 | MRA_3771 |
| 0.498523 | MRA_1582 |
| 0.50075 | MRA_2455 |
| 0.501182 | MRA_3029 |
| 0.501773 | MRA_1276 |
| 0.502318 | MRA_1862 |
| 0.502727 | MRA_1636 |

*Note*: The list arranged from top bottom based on less similarity with the human probiotic’s genomes.
